# Supply chain decision based on green investment subsidy and risk aversion

**DOI:** 10.1371/journal.pone.0293924

**Published:** 2023-11-06

**Authors:** Pengfei Liu, Shasha Yu, Zigan Lin

**Affiliations:** 1 School of Transportation Engineering, Changsha University of Science and Technology, Changsha, Hunan, China; 2 The Tobacco Monopoly Bureau of Pingjiang County (Branch Office), Yueyang, Hunan, China; Central Queensland University, AUSTRALIA

## Abstract

Considering the risk aversion characteristics of supply chain members, how to effectively design the government subsidy strategy and green supply chain strategy is a realistic and urgent issue. Regarding this, we optimize and compare four three-stage Stackelberg game models between government and a two-echelon green supply chain, namely both manufacturer and retailer risk neutral (BN), manufacturer risk aversion while retailer risk neutral (MA), retailer risk aversion while manufacturer risk neutral (RA) and both manufacturer and retailer risk aversion (BA). The government as the leader decides the subsidy rate of green input cost with the goal of maximizing social welfare; the manufacturer as the first follower makes decisions on product greenness and wholesale price to maximize its own interests; and the retailer as the second follower determines retail prices to maximize its own interests. Employing mathematical reasoning and numerical simulation investigate thoroughly the effects of the government subsidies and the members’ risk aversion. Results indicate that an appropriate government subsidy investment has a positive effect on optimal decisions and related benefits. Risk aversion is in favor of improvement of product greenness and social welfare while reduction of retail price. With the increase of manufacturer risk aversion, green subsidy investment rate and retailer expected revenue increase; on the contrary, the wholesale price and manufacturer expected revenue decrease. With the increase of retailer risk aversion, the wholesale price and manufacturer expected revenue increase, while green subsidy investment rate and retailer expected revenue decrease. In the model of BN, product greenness and social welfare are the lowest, while retail price is the highest. BA is opposite to BN. In the model of RA, green subsidy investment rate and retailer expected revenue the lowest, while wholesale price and manufacturer expected revenue the highest. RA is opposite to MA. The government should formulate appropriate subsidy policies to encourage manufacturers to produce green products and raise consumers’ green awareness. Enterprises should control their own risk aversion and assess the risk aversion of the other party reasonably.

## 1. Introduction

The growing problems of global warming, environmental pollution, energy consumption and resource shortages have threatened the world, which has led green supply chain management to become particularly important. Implementing green supply chain has an important goal of minimizing environmental impacts and increasing resource efficiency. “Made in China 2025” puts forward the need to fully execute green manufacturing as well as build an efficient and clean green manufacturing system. Three companies, Haier, Gree and Shanghai General Motors have continuously launched green products to promote green development, to enhance their corporate image in environmental protection and to gain competitive advantages. “Carbon Emissions Peak by 2030” and “Carbon Neutral by 2060” require the cleanness and greening of Chinese industrial chain. Datang Group Co. Ltd strives to build a world-class energy supplier of “green and low-carbon, multi-energy complement, efficient collaboration and digital wisdom” to realize green transformation and sustainable development.

Ecological issues, due to the use of non-green products, become a serious concern. All societal parties including individuals, corporations and governments are responsible for mitigating the ecological issues. The green products’ effect on energy saving and environmental pollution reduction is obvious. Consumers pay increasing attention to environmental issues. Especially, some consumers with low green preferences are willing to buy green products. Recent surveys indicate that about 26 percent of Europeans regularly show interest in purchasing green products and about 66 percent of consumers world-wide declare their willingness to pay for green products [1]. Green products not only attract more environmental consumers but also affect the cost of the enterprise. Most of green products require companies to pay more costs on design, research, and development, which will higher enterprises’ investment costs. Such significant costs obviously lower the enterprises’ enthusiasm to implement green projects. Without government regulations, supply chain players are unlikely to be motivated to make appropriate green improvements. Effective government policies can regulate, guide, and adjust the production and operation strategies of these enterprises. In order to promote the implementation of green activities, the government would take all kinds of measures, among which subsidies were the most common one [2]. China’s energy-saving product project was launched in 2009, aiming to compensate the spending by manufacturer on green technology and the procurement of environment friendly components [3]. In a green campaign for electric vehicles, the European Commission invested EUR41.8 million in 2012. China has supported 300 billion RMB in subsidies since 2013 and will continue to do so. According to a report in 2015 issued by the Overseas Development Institute in the UK and International Oil Exchange Organization, G20 nations spent $452 billion a year in subsidy investment. Implementation of subsidies has improved green products’ competitiveness in the entire markets. Government subsidies have greatly advanced environment sustainable development and become a valid measure to support green products’ development [4].

Governments make full use of subsidies to encourage green production. However, enterprises are risk averse, and they seek to maximize their own benefit. This behavior implies that manufacturing enterprises need an active guidance from national governments for their environmental efforts. Considering the characteristics of risk aversion of supply chain members, how to effectively design the government subsidy strategy and green supply chain management strategy is a realistic and urgent issue.

Given that, we establish four three-stage game models between government and a two-echelon green supply chain that consists of one manufacturer and one retailer, considering the supply chain members’ risk aversion characteristics and consumers’ green preference with market demand. In terms of the optimization theory and Stackelberg game model, government decides first the subsidy rate of green cost with the goal of maximizing social welfare and then the manufacturer makes decisions sequentially on product greenness and wholesale price to maximize its own interests, and following the retailer determines retail prices to maximize its own interests with four models, i.e., both manufacturer and retailer risk neutral(BN), manufacturer risk aversion while retailer risk neutral(MA), retailer risk aversion while manufacturer risk neutral(RA) and both manufacturer and retailer risk aversion(BA). By means of mathematical reasoning and numerical simulation, the influence mechanism of the government’s green investment subsidies, manufacturer’s and retailer’s risk aversion characteristics on green supply chain decision-making are discussed in this paper. It can not only provide reference for green supply chain enterprises to make reasonable decisions when coping with the risk aversion characteristics of the other party, but also provide theoretical support for relevant government departments to determine the appropriate subsidy intensity when green supply chain enterprises have the risk aversion characteristics.

This paper presents the answers to the following questions:

(1) Within these four models, what kind of roll does the one with the manufacturer and retailer risk aversion / neutral characteristics play in the decision-making differences in green supply chain?

(2) In green supply chain, how does government subsidy investment influence optimal decisions, expected returns and social welfare?

(3) How does risk aversions affect optimal decisions, expected returns and social welfare in green supply chain?

The main innovation of this paper are as follows. Firstly, most of the previous literature considered only single perspective of either government subsidies or risk aversion characteristics of green supply chain players. However, there is a lack of research on integrating both into the green supply chain. This paper comprehensively considers both government subsidies and green supply chain members’ risk aversion for the purpose of making the model closer to reality. Secondly, most studies only take government subsidy as an exogenous parameter and do not discuss how to determine the optimal proportion of government green subsidy, and supply chain decision was applied two-stage game. In this paper, we take government investment subsidy as an endogenous variable which optimizes in the goal of maximizing social welfare. Decision-making is adopted a three-stage game dominated by the government. Thirdly, most literatures only consider the risk aversion behavior of one party. However, the addition of "greenness" in green supply chain makes the market demand more volatile, and the risk aversion of supply chain members becomes stronger. It is necessary to incorporate the both manufacturer and retailer risk aversion characteristic into the decision-making analysis of green supply chain. We construct four three-stage Stackelberg game models with green investment subsidy, compare and analyze the influence of risk aversion characteristics on the proportion of government green subsidies, decision variables and the utility value of green supply chain members. Besides, we discuss whether the government green subsidy strategy will change these influences. Fourthly, we consider more comprehensive factors, such as the green preference sensitivity of consumers, the per coefficient of retail price on market demand, unit cost of production, etc. which make the green supply chain decision model with risk aversion more practical and it is closer to the actual market situation and can be used as a guide in the market.

The remainder of this paper is structured as follows: Section 2 summarizes the literature; Section 3 presents model description and basic assumptions; Section 4 establishes four game models and obtains the corresponding equilibrium solutions; Section 5 compares and analyzes the four game models’ solutions; Section 6 conducts the numerical analysis to gain more management insights; Section 7 states the conclusions.

## 2. Literature review

### 2.1 Government subsidies for green supply chain

With the rapid development of green supply chain management theory and practice, how to effectively carry out green supply chain management has become a hot topic in academic research and practice. Government can support companies to become greener and to develop environment friendly processes and products with greening subsidies [5]. The research on government subsidy strategies for green supply chain focuses on the subsidy object, subsidy method and their influences. Government green subsidies to manufacturer are now widespread because manufacturer need more green investment to produce green products. Despite government subsidies to manufacturer, retailer become the beneficiaries. Additionally, as the level of government subsidies increases, the green level of products can also increase. However, the supply chain and manufacturer do not always benefit from government subsidies [6]. As for the advantages of government green subsidies, Sheu and Chen [7] revealed that government green subsidies may enhance supply chain competitiveness as well as increase consumers’ social welfare. Xue et al. [8] indicated that government subsidies play an active role in the improvement of energy-saving level of products and social welfare could be promoted. Guo et al. [9] concluded that the subsidy policy is not only the most effective way but also very necessary condition to maximize the expected social welfare and expected utility. Yu et al. [10] demonstrated that a good subsidy policy can create more expected utility for manufacturer and save the subsidy investment by government at the same time. Li et al. [11] confirm that government green subsidies can effectively promote green technology innovation as well as ecological environmental protection. Wang and Zhao et al. [12] explored that “too high” or “too low” government subsidies for re-manufacturer subsidies make re-manufacturer compete with manufacturer, while intermediate subsidies cause two members to cooperation. In the duopoly market, the government should impose higher environmental taxes yet provide lower subsidies for electric vehicles [13]. Mitra et al. [14] compared and analyzed the impact of government subsidies to re-manufacturer, to manufacturer and to both respectively. Abhijit et al. [15] revealed that government subsidies can reduce the cost of enterprises. Meng and Li [16] explored that government subsidies are beneficial to manufacturers and have little impact on retailers. Recently, three types of innovative subsidy scenarios were analyzed by Meng and Wang et al. [17], such as government subsidizes to both manufacturer and upstream supplier, to only core manufacturer, to core manufacturer and to core enterprise subsidies upstream supplier, in the condition of consumer green and non-green heterogeneity. In this paper, the effect of subsidies only to manufacturer with green supply chain strategies will be considered. Hence, the literature regarding government green subsidies to retailer/consumer is ignored.

In view of subsidy investment, one question is subsidies to whom, and the other is how to subsidize. Hussain et al. [18] ensured the realization of green technology as well as that enterprises will not adopt green technology innovation without subsidies. Yu et al. [10] investigated the decision-making in light of government subsidies to manufacturer’ greenness of products and discovered that consumers’ environmental awareness improvement can encourage manufacturer to produce more green products. When the governments provide subsidies for the development of green technology, the social welfare will always increase as the industry develops [19]. Yang et al. [20] deemed that subsidizing energy-saving products can guide consumers to buy low-carbon products and producers to produce low-carbon products. Shi et al. [21] believed that unit product subsidies are more effective than one-time subsidies to manufacturer. Wang and Chang et al. [22] proposed that subsidizing green R&D innovation costs of manufacturer will encourage them to increase their green efforts, Zhong et al. [23] showed that innovation subsidy can always achieve a better social welfare than quantity subsidy. while Bai et al. [24] manifested that government R&D subsidies can be effectively promote the green innovation of energy-intensive enterprises. Wang et al. [25] pinpointed that green government and green insurance subsidies can promote enterprises’ green innovation, though direct government subsidies will pose a greater risk for green innovation. Lately, Li et al. [26] showed that green product subsidy is not always the best option for government. Nevertheless, green innovation subsidy is always better than green product subsidy for maximizing subsidy efficiency.

In addition to subsidy strategies, the government also implements carbon emission reduction and carbon tax on the green supply chain. Xu et al. [27] constructed a supply chain in which the government subsidizes supply chain participants and collects carbon tax and analyzed the impact of horizontal integration in the supply chain. Hussain et al. [28] established a model measuring the best behavior of green technology investment to reduce carbon emissions. Some scholars study the game of supply chain participants’ weaving by constructing a supply chain with a preference for green products. Rahmani and Yavari [29] studied the pricing strategy of green supply chain under the interruption of market demand and found that when the market scale was destroyed or the greening cost was reduced, the optimal price in both decision-making structures would increase. Das et al. [30] analyzed the greening level of products and other factors and thought that supply chain members could really compromise on the marginal expected utility target to achieve sustainable development. The research results may help supply chain members to implement more sustainable development plans. Appropriate government subsidies including subsidy object and subsidy method can promote the greening of supply chain products. This paper discusses the decision-making problem of subsidizing manufacturers’ green investment.

### 2.2 Green supply chain decision with government subsidies via game theory

More attention is paid to either the governments’ decision-making or the relationship between the government and the green supply chain enterprises’ decision-making. Game theory has proven to be an effective analytical tool to consider various government policies on subsidies for green supply chain. Lou et al.[31] studied government green subsidies of the two-echelon supply chain and the optimal strategies problems of manufacturer and retailer. Sheu and Chen [7] employed a three-stage game theory model to analyze the influence of government subsidies on the competition within a green supply chain. Hossein et al. [32] analyzed model with different government subsidy strategies in a supply chain manufacturing and selling a green product. Peng [33] constructed a model to obtain both the price strategy and the optimal production quantity given the subsidies for new energy vehicles. Wang et al. [34] adopted the game theory approach to investigate the role of subsidy policy and government fines on the new energy vehicles supply chain, and the result indicated that government subsidies influence the vehicle manufacturer’ choices. Sinayi et al. [35] compared and analyzed the game decisions of supply chain without government subsidy and with emission reduction level subsidy. Li et al. [11] established a game model between manufacturer and retailer, exploring the influence of government subsidies and substitute subsidies on environment friendly products. Game theory was applied by He et al. [36] and they reached to an optimum pricing decisions in different channel structure scenarios with government subsidy. Guo et al. [9] developed a game model composed of suppliers, manufacturer, and governments. Based on this model, they analyzed the impact of government subsidies on social welfare and on the supply chain members’ expected utility. Hafezalkotob et al. [37] designed a Stackelberg game model with the government as the leader and compared the impact of government subsidy policy on both ordinary and green supply chains. Zhao and Zeng et al. [38] studied how the expected utility is transferred and analyzed the influence of different government subsidies on the competitiveness of the supply chain. Sinayi et al. [35] set up a two-tier supply chain model with the government as the leader as well and discussed a government utility function’s effect on the supply chain members’ expected utility as well as on the product’s green degree. Stackelberg game was applied to propose a green supply chain model with government subsidies and a model without government subsidies but with a dual-channel structure, in which consumers have both green preference and channel preference [16]. A three-stage Stackelberg game model was developed and analytically remodeled with three different government subsidy strategies by Khosroshahi [39], such as retail price subsidy, transparent cost subsidy and manufacturing cost subsidy in a supply chain of manufacturing and selling green products. Most of the extant literature regarding government subsidies via game theory apply a two-stage game, taking government subsidy as an exogenous parameter rather than decision variables. We utilize general three-stage Stackelberg game method to optimize the green supply chain decisions with government subsidies.

### 2.3 Green supply chain decision with risk aversion

The literature above employed general game models to study government and enterprise behavioral strategies with subsidy policies, which ignored the decision-makers’ behavioral factors. In fact, market volatility may make supply chain decision makers risk averse. Because of consumer demand for various products, there is uncertainty in the sales volume of green products. The impact of risk in the supply chain is particularly significant. Therefore, the research conclusion has great limitations. However, with respect to the game theory model’s assumption of perfect rational behavior, the risk averse behavior hypothesis of members appears to have more realistic significance. Jiang et al. [40] analyzed the impact of manufacturer’s risk aversion on the decision-making of green supply chain members. Cao et al. [41] constructed a game model between a risk neutral manufacturer and N risk averse distributors to explore the impact of the risk aversion on the pricing of green products, order quantity and the income of supply chain members. Han et al. [42] studied that the order quantity and sales effort level are determined by risk-averse retailers under the conditional value at risk (CVaR) criterion, and the retailers make sales effort and bear the effort cost. Raza [43] developed dual-channel green supply chain models that the manufacturer and retailer are assumed to be risk averse and used the mean-variance criterion to measure the risk aversion. Chen et al.[44] developed decentralized decision-making models under fairness concern and risk aversion, and derived the optimal strategy for each member with a Stackelberg game in which the manufacturer acts as the leader. Xie and Hou et al. [45] showed that when the manufacturer’s innovation cost coefficient is relatively low, the more expected utility per-unit production subsidy may be abandoned due to its performance instability. Hou and Wang et al. [46] considered one manufacturer who produces a green product and distributes the product through one physical retailer. The results show that the retailer’s risk-averse behavior can alleviate the expected utility loss. Only risk aversion is considered without incorporating subsidy strategies in most of the existing literature on green supply chain decision with risk aversion.

The above-mentioned literature studied government green subsidies or member risk aversion from a single perspective but did not include them in the research of green supply chain at the same time. Only a few scholars have studied the impact of risk aversion characteristics on green decision-making with government subsidies. Cohen et al. [47] studied the impact of government green technology subsidies on participants’ decision-making when demand is uncertain. Xie et al. [48] considered the balance between the risk and the return of the main body of the supply chain in two different supply chain structures, and took the green supply chain of household appliances in China as an example to discuss the relevant issues in the supply chain.

Generally, We comprehensively consider both government subsidies and green supply chain members’ risk aversion, analyze the impact of the risk aversion characteristics on the proportion of government green subsidies, decision variables and utility value of green supply chain members, and discuss whether the government green subsidy strategy changes these effects.

## 3. Model description and assumptions

We establish a game model between the government and a two-echelon green supply chain composed of a manufacturer and a retailer. The government aims to maximize social welfare, while the manufacturer and retailer aim to maximize its own interests. Firstly, government decides subsidy proportion λ of the green investment. Secondly, manufacturer optimizes product greenness e and wholesale price a. Finally, retailer decides product sale price p. The market demand of green products is affected by its own price and product green degree. Consumers have preferences for green products and will pursue products with lower price and higher green degree. Market demand function is: D = α−βp+γe+ε, where α indicates the potential market demand; β *and* γ represent the influence of market demand on product retail price and consumer green preference coefficient respectively (β,γ>0). When manufacturer produces green products, the unit production cost c remains unchanged. But manufacturer needs to increase additional green investment C, which is a one-time investment. Supposing that C and product green degree e are quadratic, then C = ηe^2^/2 by referring to Sheu et al. [7].

For research, we make the following assumptions:

ε is the uncertainty of market demand and obeys the normal distribution with the mean value 0 and the variance σ^2^, i.e., ε∈N(0, σ^2^).

η is the cost coefficient of green input. We assume 16βη>30γ^2^.

In order to encourage manufacturer to produce green products, the government will make the investment of green cost in proportion λ financial subsidies to manufacturer, where λ∈(0,1).

Social welfare can be added linearly. Social welfare (Π_sw_) = Consumer surplus + Producer surplus − Government subsidies by referring to Sheu et al. [7].

φ_i_ = μ_i_σ^2^(i = m,r) indicates the risk aversion of manufacturer and retailer by referring to Bai et al. [49] and Zou et al. [50]. The larger the value of φ is, the more the supply chain participants afraid of the risks are. When φ_i_ = 0, the supply chain participants are risk neutral. In order to satisfy the following requirements, it is assumed that 9φ_m_−β>0.

## 4. Game model

### 4.1 A decision model in which both manufacturer and retailer are risk neutral (BN)

Note that both the manufacturer and the retailer are considering risk aversion. We consider using the mean variance method to characterize their respective utility expected utility (U).i.e. U = E(Π)−μ*Var*(Π) = E(Π)−μ*E*[Π−*E*(Π)]^2^. According to what mentioned above, the decision functions of the manufacturer, retailer and government are as follows:

$$U(\Pi_{m1})=E(\Pi_{m1})=(\omega -c)(\alpha -\beta p+\gamma e)-(1-\lambda )\eta e^{2}/2$$ (1)

$$U(\Pi_{r1})=E(\Pi_{r1})=(p-\omega )(\alpha -\beta p+\gamma e)$$ (2)

$$\Pi_{sw1}=\frac{{\left(\alpha -\beta p+\gamma e\right)}^{2}}{2\beta }+(p-c)(\alpha -\beta p+\gamma e)-\eta e^{2}/2$$ (3)

The second derivative of retailer’s expected utility formula for p is $\frac{\partial^{2}U(\Pi_{r1})}{\partial p^{2}}=-2b<0$. Therefore, U(Π_r1_) is a strictly concave function of p. Find the first partial derivative of (2) to p and make it equal to 0:

p=(α+βω+γe)/2β (4)

Substituting Eq (4) into Eq (1), finding Hessian matrix of U(Π_m1_):

$$|H_{1}|=\left|\begin{array}{cc} -\beta & \frac{\gamma }{2}\\ \frac{\gamma }{2} & -\eta (1-\lambda ) \end{array}\right|$$

The first order principal sub-matrix of the Hesse matrix of U(Π_m1_) is −β, as long as the determinant $|H_{1}|=\beta \eta (1-\lambda )-\frac{\gamma^{2}}{4}>0$. There is negative definite matrix. So U(Π_m1_) is a joint concave function of ω and e. Similar to Rahmani et al. [29] and Das et al. [30].

Use reverse induction to solve the problem, substitute Eq (4) into Eq (1), find the first partial derivatives of ω and e and make them equal to 0. The wholesale price and green degree can be obtained by simultaneous equations. By putting it into Eq (4) we will get the retail price.

$$\Omega_{1}=\frac{2\eta (1-\lambda )(\alpha +\beta c)-c\gamma^{2}}{4\beta \eta (1-\lambda )-\gamma^{2}}$$ (5)

$$E_{1}=\frac{\gamma (\alpha -\beta c)}{4\beta \eta (1-\lambda )-\gamma^{2}}$$ (6)

$$p_{1}=\frac{\eta (1-\lambda )(3\alpha +\beta c)-c\gamma^{2}}{4\beta \eta (1-\lambda )-\gamma^{2}}$$ (7)

Substitute Eqs (6) and (7) into the social welfare formula, find the first partial derivative of λ and make it equal to 0. The optimal subsidy ratio is $\lambda_{1}^{*}=3/7$. By substituting the value of a $\lambda_{1}^{*}$ into Eqs (5)–(7). The optimal wholesale price, product greenness and retail price can be obtained:

$$\omega_{1}^{*}=\frac{8\eta (\alpha +\beta c)-7c\gamma^{2}}{16\beta \eta -7\gamma^{2}}$$ (8)

$$e_{1}^{*}=\frac{7\gamma (\alpha -\beta c)}{16\beta \eta -7\gamma^{2}}$$ (9)

$$p_{1}^{*}=\frac{4\eta (3\alpha +\beta c)-7c\gamma^{2}}{16\beta \eta -7\gamma^{2}}$$ (10)

Substitute the optimal subsidy ratio and Eqs (8)–(10) into Eqs (1)–(3), the maximum utility and expected social welfare of manufacturer and retailer can be obtained:

$${U(\Pi_{m1})}^{*}=\frac{2\eta {\left(\alpha -\beta c\right)}^{2}}{16\beta \eta -7\gamma^{2}}$$ (11)

$${U(\Pi_{r1})}^{*}=\frac{16\beta \eta^{2}{\left(\alpha -\beta c\right)}^{2}}{{\left(16\beta \eta -7\gamma^{2}\right)}^{2}}$$ (12)

$${E(\Pi_{sw1})}^{*}=\frac{7\eta {\left(\alpha -\beta c\right)}^{2}}{2(16\beta \eta -7\gamma^{2})}$$ (13)

### 4.2 A decision model with manufacturer as risk neutral and retailer as risk averse (RA)

The function of decision-retailer is:

$$U(\Pi_{r2})=E(\Pi_{r2})-\mu_{r}Var(\Pi_{r2})=E(\Pi_{r2})-\mu_{r}\sigma^{2}{\left(w-c\right)}^{2}=E(\Pi_{r2})-\varphi_{r}{\left(w-c\right)}^{2}=(p-\omega )(\alpha -\beta p+\gamma e)-\varphi_{r}{\left(p-\omega \right)}^{2}$$ (14)

The function of decision-manufacturer is the same as the Eq (1), i.e. U(Π_m2_) = U(Π_m1_), and the function of decision-government is the same as the Eq (3), Π_sw2_ = Π_sw1_.

Hessian matrix of U(Π_m2_) is:

$$|H_{2}|=\left|\begin{array}{cc} -\frac{\beta (\beta +2\varphi_{r})}{\beta +\varphi_{r}} & -\frac{\beta \gamma }{2\beta +2\varphi_{r}}+\gamma \\ -\frac{\beta \gamma }{2\beta +2\varphi_{r}}+\gamma & -\eta (1-\lambda ) \end{array}\right|$$

To ensure that U(Π_m2_) is a joint concave function with respect to ω and e, the first order principal sub-matrix of the Hesse matrix of U(Π_m2_) is $-\frac{\beta (\beta +2\varphi_{r})}{\beta +\varphi_{r}}$, as long as the determinant $|H_{2}|=\frac{\eta \beta (\beta +2\varphi_{r})(1-\lambda )}{\beta +\varphi_{r}}-{\left(\gamma -\frac{\beta \gamma }{2\beta +2\varphi_{r}}\right)}^{2}>0$. According to part 4.2, the optimal decision value $\omega_{2}^{*},e_{2}^{*},p_{2}^{*},{U(\Pi_{m2})}^{*},{U(\Pi_{r2})}^{*},{\Pi_{sw2}}^{*}$ can be obtained.

### 4.3 A decision model with manufacturer as risk averse and retailer as risk neutral (MA)

The function of decision-manufacturer is:

$$U(\Pi_{m3})=E(\Pi_{m3})-\mu_{m}Var(\Pi_{m3})=E(\Pi_{m3})-\mu_{m}\sigma^{2}{\left(w-c\right)}^{2}=E(\Pi_{m3})-\varphi_{m}{\left(w-c\right)}^{2}=(\omega -c)(\alpha -\beta p+\gamma e)-\frac{(1-\lambda )\eta e^{2}}{2}-\varphi_{m}{\left(\omega -c\right)}^{2}$$ (15)

The function of decision-retailer is the same as the Eq (2), i.e, U(Π_r3_) = U(Π_r1_), and the function of decision-government is the same as Π_sw1_, i.e., Π_sw3_ = Π_sw1_.

Hessian matrix of U(Π_m3_) is:

$$|H_{3}|=\left|\begin{array}{cc} -\beta -2\varphi_{m} & \frac{\gamma }{2}\\ \frac{\gamma }{2} & -\eta (1-\lambda ) \end{array}\right|$$

To ensure that U(Π_m3_) is a joint concave function with respect to ω and e, the first order principal sub-matrix of the Hesse matrix of U(Π_m3_) is −β−2φ_m_, as long as the determinant $|H_{3}|=\eta (\beta +2\varphi_{m})(1-\lambda )-\frac{\gamma^{2}}{4}>0$. The optimal decision value $\omega_{3}^{*},e_{3}^{*},p_{3}^{*},{U(\Pi_{m3})}^{*},{U(\Pi_{r3})}^{*},{\Pi_{sw3}}^{*}$ can be obtained.

### 4.4 A decision model in which both manufacturer and retailer are risk averse (BA)

The function of decision-maker is:

$$U(\Pi_{m4})=E(\Pi_{m4})-\mu_{m}Var(\Pi_{m4})=E(\Pi_{m4})-\mu_{m}\sigma^{2}{\left(w-c\right)}^{2}=E(\Pi_{m4})-\varphi_{m}{\left(w-c\right)}^{2}=(\omega -c)(\alpha -\beta p+\gamma e)-\frac{(1-\lambda )\eta e^{2}}{2}-\varphi_{m}{\left(\omega -c\right)}^{2}$$ (16)

$$U(\Pi_{r4})=E(\Pi_{r4})-\mu_{r}Var(\Pi_{r4})=E(\Pi_{r4})-\mu_{r}\sigma^{2}{\left(w-c\right)}^{2}=E(\Pi_{r4})-\varphi_{r}{\left(w-c\right)}^{2}=(p-\omega )(\alpha -\beta p+\gamma e)-\varphi_{r}{\left(p-\omega \right)}^{2}$$ (17)

the function of decision-government is the same as Π_sw1_, i.e. Π_sw4_ = Π_sw1_

Hessian matrix of U(Π_m4_) is:

$$|H_{4}|=\left|\begin{array}{cc} -\frac{\beta (\beta +2\varphi_{r})}{\beta +\varphi_{r}}-2\varphi_{m} & -\frac{\beta \gamma }{2\beta +2\varphi_{r}}+\gamma \\ -\frac{\beta \gamma }{2\beta +2\varphi_{r}}+\gamma & -\eta (1-\lambda ) \end{array}\right|$$

To ensure that U(Π_m4_) is a joint concave function with respect to ω and e, the first order principal sub-matrix of the Hesse matrix of U(Π_m4_) is $-\frac{\beta (\beta +2\varphi_{r})}{\beta +\varphi_{r}}-2\varphi_{m}$, as long as the determinant $|H_{4}|=\eta (1-\lambda )(\frac{\beta (\beta +2\varphi_{r})}{\beta +\varphi_{r}}-2\varphi_{m})-{\left(\gamma -\frac{\beta \gamma }{2\beta +2\varphi_{r}}\right)}^{2}>0$. The optimal decision value $\omega_{4}^{*},e_{4}^{*},p_{4}^{*},{U(\Pi_{m4})}^{*},{U(\Pi_{r4})}^{*},{\Pi_{sw4}}^{*}$ can be obtained.

The results of the four decision models are summarized in the following Table 1.

**Table 1:** The decision results of four models.

	BN (φ_m_ = 0, φ_r_ = 0)	RA (φ_m_ = 0, φ_r_ > 0)	MA (φ_m_ > 0, φ_r_ = 0)	BA (φ_m_ > 0, φ_r_ > 0)
λ*	$\frac{3}{7}$	$\frac{3\beta +2\varphi_{r}}{7\beta +6\varphi_{r}}$	$\frac{48{\varphi_{m}}^{2}+32\beta \varphi_{m}+3\beta^{2}}{\left(7\beta +12\varphi_{m})(\beta +4\varphi_{m}\right)}$	$\frac{C_{1}+\beta (3\beta^{3}{+C}_{2})(\beta +2\varphi_{r})}{C_{1}+\beta (7\beta^{3}+C_{3})(\beta +2\varphi_{r})}$
ω*	$\frac{8\eta (\alpha +\beta c)-7c\gamma^{2}}{16\beta \eta -7\gamma^{2}}$	$\frac{8\alpha \eta {\left(\beta +\varphi_{r}\right)}^{2}+A_{2}}{A_{1}}$	$\frac{8\alpha \beta \eta (\beta +2\varphi_{m})+B_{2}}{B_{1}}$	$\frac{8\alpha \beta \eta (\beta +2\varphi_{r})(\beta +\varphi_{r})C_{4}+cC_{6}(\beta +\varphi_{r})(8\beta \eta C_{4}-\gamma^{2}C_{5})}{16\beta \eta {C_{4}}^{2}-\gamma^{2}C_{5}C_{6}(\beta +\varphi_{r})}$
e*	$\frac{7\gamma (\alpha -\beta c)}{16\beta \eta -7\gamma^{2}}$	$\frac{\gamma (\beta +2\varphi_{r})(\alpha -\beta c)(7\beta +6\varphi_{r})}{A_{1}}$	$\frac{\gamma (\alpha -\beta c)(7\beta +12\varphi_{m})(\beta +4\varphi_{m})}{B_{1}}$	$\frac{\gamma C_{5}(\beta +2\varphi_{r})(\alpha -\beta c)(2(\beta +2\varphi_{m})(\beta +\varphi_{r})-\beta^{2})}{16\beta \eta {C_{4}}^{2}-\gamma^{2}(\beta +\varphi_{r})C_{5}C_{6}}$
p*	$\frac{4\eta (3\alpha +\beta c)-7c\gamma^{2}}{16\beta \eta -7\gamma^{2}}$	$\frac{4\alpha \eta (3\beta +2\varphi_{r})(\beta +\varphi_{r})+A_{3}}{A_{1}}$	$\frac{4\alpha \eta (3\beta +4\varphi_{m})(\beta +2\varphi_{m})+B_{3}}{B_{1}}$	$\frac{4\alpha \beta \eta C_{4}C_{7}+cC_{6}(\beta +2\varphi_{r})(4\beta \eta C_{4}-\gamma^{2}C_{5})}{16\beta \eta {C_{4}}^{2}-\gamma^{2}C_{5}C_{6}(\beta +\varphi_{r})}$
U(Π_m_)*	$\frac{2\eta {\left(\alpha -\beta c\right)}^{2}}{16\beta \eta -7\gamma^{2}}$	$\frac{2\eta {\left(\alpha -\beta c\right)}^{2}(\beta +\varphi_{r})(\beta +2\varphi_{r})}{A_{1}}$	$\frac{2\beta \eta {\left(\alpha -\beta c\right)}^{2}(\beta +2\varphi_{m})}{B_{1}}$	$\frac{2\beta \eta (\beta +2\varphi_{r}){\left(\alpha -\beta c\right)}^{2}C_{4}}{16\beta \eta {C_{4}}^{2}-\gamma^{2}C_{5}C_{6}(\beta +\varphi_{r})}$
U(Π_r_)*	$\frac{16\beta \eta^{2}{\left(\alpha -\beta c\right)}^{2}}{{\left(16\beta \eta -7\gamma^{2}\right)}^{2}}$	$\frac{16\beta^{2}\eta^{2}{\left(\beta +\varphi_{r}\right)}^{3}{\left(\alpha -\beta c\right)}^{2}}{{A_{1}}^{2}}$	$\frac{16\beta \eta^{2}{\left(\alpha -\beta c\right)}^{2}{\left(\beta +2\varphi_{m}\right)}^{2}{\left(\beta +4\varphi_{m}\right)}^{2}}{{B_{1}}^{2}}$	$\frac{16\beta^{2}\eta^{2}{\left(\alpha -\beta c\right)}^{2}(\beta +\varphi_{r})C_{8}}{{\left(16\beta \eta {C_{4}}^{2}-\gamma^{2}(\beta +\varphi_{r})\right)}^{2}{C_{5}}^{2}{C_{6}}^{2}}$
Π_sw_*	$\frac{7\eta {\left(\alpha -\beta c\right)}^{2}}{2(16\beta \eta -7\gamma^{2})}$	$\frac{\eta {\left(\alpha -\beta c\right)}^{2}(7\beta +6\varphi_{r})(\beta +2\varphi_{r})}{2A_{1}}$	$\frac{\eta {\left(\alpha -\beta c\right)}^{2}(7\beta +12\varphi_{m})(\beta +4\varphi_{m})}{{2B}_{1}}$	$\frac{\eta C_{5}{\left(\alpha -\beta c\right)}^{2}(\beta +2\varphi_{r})((\beta +2\varphi_{r})(\beta +2\varphi_{m})+2\beta \varphi_{m})}{2(16\beta \eta {C_{4}}^{2}-\gamma^{2}C_{5}C_{6}(\beta +\varphi_{r}))}$

Where $A_{1}=16\eta \beta {\left(\beta +\varphi_{r}\right)}^{2}-\gamma^{2}(7\beta +6\varphi_{r})(\beta +2\varphi_{r});A_{2}=c(8\eta \beta {\left(\beta +\varphi_{r}\right)}^{2}{-\gamma }^{2}(7\beta +6\varphi_{r})(\beta +2\varphi_{r}));$

$A_{3}=c(\beta +2\varphi_{r})(4\eta \beta (\beta +\varphi_{r}){-\gamma }^{2}(7\beta +6\varphi_{r}));B_{1}=16\beta \eta {\left(\beta +2\varphi_{m}\right)}^{2}-\gamma^{2}(7\beta +12\varphi_{m})(\beta +4\varphi_{m});$

$B_{2}=c(\beta +4\varphi_{m})(8\beta \eta (\beta +2\varphi_{m})-\gamma^{2}(7\beta +12\varphi_{m}));B_{3}=c(\beta +4\varphi_{m})(4\eta \beta (\beta +2\varphi_{m})-\gamma^{2}(7\beta +12\varphi_{m}))$;

$C_{1}=16{\varphi_{m}}^{2}(3\beta +2\varphi_{r}){\left(\beta +\varphi_{r}\right)}^{2};C_{2}=4{\varphi_{r}}^{2}(\beta +6\varphi_{m})+8\beta (4\beta \varphi_{m}+\beta \varphi_{r}+7\varphi_{m}\varphi_{r});$

$C_{3}=4{\varphi_{r}}^{2}(3\beta +8\varphi_{m})+\beta (5\beta \varphi_{m}+10\beta \varphi_{r}+18\varphi_{m}\varphi_{r});C_{4}=(\beta +\varphi_{r})({\left(\beta +2\varphi_{m}\right)}^{2}-2\varphi_{m}\varphi_{r});$

$C_{5}=\beta (7\beta +12\varphi_{m})(\beta +2\varphi_{r})+\varphi_{r}(4(3\beta +2\varphi_{m})(\varphi_{m}-\beta )-18\beta^{2});C_{6}={\left(\beta +2\varphi_{m}\right)}^{2}+2\beta \varphi_{m};$

$C_{7}=4{\left(\beta +\varphi_{m}\right)}^{2}+{\left(\beta +2\varphi_{r}\right)}^{2}-2\beta^{2};C_{8}=4(2\varphi_{m}\varphi_{r}+\beta \varphi_{m})(2\beta \varphi_{m}+3\beta^{2})+\beta^{2}(2(\beta +\varphi_{r})(2\beta +3\varphi_{m})-3\beta^{2})+4{\varphi_{r}}^{2}(2{\varphi_{m}}^{2}+3\beta \varphi_{m}+\beta^{2})$.

According to the decision results of the four decision models in Table 1, the first derivatives of the parameters are obtained respectively. The results are shown in the Table 2.

**Table 2:** The influence of parameters in game model (i = 1,2,3,4).

	β	γ	η	φ_m_	φ_r_
λ*	$\frac{\partial \lambda_{1}^{*}}{\partial \beta }=0;\frac{\partial \lambda_{2}^{*}}{\partial \beta }>0;$ $\frac{\partial \lambda_{3}^{*}}{\partial \beta }<0;\frac{\partial \lambda_{4}^{*}}{\partial \beta }<0$	$\frac{\partial \lambda_{i}^{*}}{\partial \gamma }=0$	$\frac{\partial \lambda_{i}^{*}}{\partial \eta }=0$	$\frac{\partial \lambda_{3}^{*}}{\partial \varphi_{m}}>0;\frac{\partial \lambda_{4}^{*}}{\partial \varphi_{m}}>0$	$\frac{\partial \lambda_{2}^{*}}{\partial \varphi_{r}}<0;\frac{\partial \lambda_{4}^{*}}{\partial \varphi_{r}}<0$
ω*	$\frac{\partial \omega_{i}^{*}}{\partial \beta }<0$	$\frac{\partial \omega_{i}^{*}}{\partial \gamma }>0$	$\frac{\partial \omega_{i}^{*}}{\partial \eta }<0$	$\frac{\partial w_{3}^{*}}{\partial \varphi_{m}}<0;\frac{\partial w_{4}^{*}}{\partial \varphi_{m}}<0;$	$\frac{\partial \omega_{2}^{*}}{\partial \varphi_{r}}>0;\frac{\partial \omega_{4}^{*}}{\partial \varphi_{r}}>0$
e*	$\frac{\partial e_{i}^{*}}{\partial \beta }<0$	$\frac{\partial e_{i}^{*}}{\partial \gamma }>0$	$\frac{\partial e_{i}^{*}}{\partial \eta }<0$	$\frac{\partial e_{3}^{*}}{\partial \varphi_{m}}>0;\frac{\partial e_{4}^{*}}{\partial \varphi_{m}}>0$	$\frac{\partial e_{2}^{*}}{\partial \varphi_{r}}>0;\frac{\partial e_{4}^{*}}{\partial \varphi_{r}}>0$
p*	$\frac{\partial p_{i}^{*}}{\partial \beta }<0$	$\frac{\partial p_{i}^{*}}{\partial \gamma }>0$	$\frac{\partial p_{i}^{*}}{\partial \eta }<0$	$\frac{\partial p_{3}^{*}}{\partial \varphi_{m}}<0;\frac{\partial p_{4}^{*}}{\partial \varphi_{m}}<0$	$\frac{\partial p_{2}^{*}}{\partial \varphi_{r}}<0;\frac{\partial p_{4}^{*}}{\partial \varphi_{r}}<0$
U(Π_m_)*	$\frac{\partial {U(\Pi_{m})}_{i}^{*}}{\partial \beta }<0$	$\frac{\partial U{\left(\Pi_{m}\right)}_{i}^{*}}{\partial \gamma }>0$	$\frac{\partial U{\left(\Pi_{m}\right)}_{i}^{*}}{\partial \eta }<0$	$\frac{\partial U{\left(\Pi_{m}\right)}_{3}^{*}}{\partial \varphi_{m}}<0;\frac{\partial U{\left(\Pi_{m}\right)}_{4}^{*}}{\partial \varphi_{m}}<0$	$\frac{\partial U{\left(\Pi_{m}\right)}_{2}^{*}}{\partial \varphi_{r}}>0;\frac{\partial U{\left(\Pi_{m}\right)}_{4}^{*}}{\partial \varphi_{r}}>0$
U(Π_r_)*	$\frac{\partial {U(\Pi_{r})}_{i}^{*}}{\partial \beta }<0$	$\frac{\partial U{\left(\Pi_{r}\right)}_{i}^{*}}{\partial \gamma }>0$	$\frac{\partial U{\left(\Pi_{r}\right)}_{i}^{*}}{\partial \eta }<0$	$\frac{\partial U{\left(\Pi_{r}\right)}_{3}^{*}}{\partial \varphi_{m}}>0;\frac{\partial U{\left(\Pi_{r}\right)}_{4}^{*}}{\partial \varphi_{m}}>0$	$\frac{\partial U{\left(\Pi_{r}\right)}_{2}^{*}}{\partial \varphi_{r}}<0;\frac{\partial U{\left(\Pi_{r}\right)}_{4}^{*}}{\partial \varphi_{r}}<0$
Π_sw_*	$\frac{\partial {U(\Pi_{sw})}_{i}^{*}}{\partial \beta }<0$	$\frac{\partial {\Pi_{sw}}_{i}^{*}}{\partial \gamma }>0$	$\frac{\partial {\Pi_{sw}}_{i}^{*}}{\partial \eta }<0$	$\frac{\partial {\Pi_{sw}}_{3}^{*}}{\partial \varphi_{m}}>0;\frac{\partial {\Pi_{sw}}_{4}^{*}}{\partial \varphi_{m}}>0$	$\frac{\partial {\Pi_{sw}}_{2}^{*}}{\partial \varphi_{r}}>0;\frac{\partial {\Pi_{sw}}_{4}^{*}}{\partial \varphi_{r}}>0$

Proposition 1: In these four decisions, with the decrease of retail price sensitivity coefficient (β) as well as green investment cost coefficient (η) and the increase of consumers’ green preference coefficient (γ), the wholesale price (ω), greenness (e), sales price (p), manufacturer’s and retailer’s expected utility (U(Π_m_), U(Π_r_)) and social welfare increase (Π_sw_).

Proposition 1 shows that when consumers have stronger preference for green products (γ) or lower sensitivity to the price of green products (β), participants in the supply chain can obtain more expected utility and social welfare increase. Meanwhile, if manufacturer can reduce the research and development costs of green products, its own expected utility can also increase, which in return leads to the expansion of retailer’s expected utility and social welfare. It shows that the government subsidy policy has an incentive effect on the manufacturers to increase the investment in green product research and development, which is consistent with the conclusions of existing literature from Sheu et al. [7]. the increase of product greenness brought by government subsidy coefficient is greater than the increase of product retail price. For consumers, government subsidy helps consumers to obtain green products with high performance-price ratio.

Proposition 2: The proportion of government subsidies has nothing to do with consumers’ green preference coefficient (γ) and green cost investment coefficient (η). When both manufacturer and retailer are risk neutral, the proportion of government subsidies is constant. When retailer is risk averse, the retail price sensitivity coefficient (β) is directly proportional to the proportion of government subsidies. When the manufacturer is risk averse or both are risk averse, the retail price sensitivity coefficient (β) is inversely proportional to the government subsidy ratio.

Proposition 2 reflects that the measures taken by supply chain participants to avoid risks will affect the proportion of government subsidies. When retailer wants to avoid risks, it will take more conservative decisions. When the retail price sensitivity coefficient of the market increases, the government will increase the proportion of government subsidies in order to ensure the expected utility of the market. Whereas, when the manufacturer is risk-averse, the manufacturer will be more conservative in decision-making for the wholesale price and the green degree. To encourage the manufacturer to produce green products, the government will increase the subsidy ratio when the retail price sensitivity coefficient decreases.

Proposition 3: In the two-decision models of manufacturer risk aversion only and both manufacturer and retailer risk aversion, the manufacturer risk aversion degree is directly proportional to the proportion of government subsidies, green degree, retailer expected utility and social welfare. It is inversely proportional to wholesale prices, retail prices and manufacturer’ expected utility.

Given that, proposition 3 can be defined that manufacturer can avoid risks by adjusting wholesale prices and greenness. When the manufacturer’s risk aversion is higher, the manufacturer will reduce the greenness of products and raise the price of goods to avoid risks, but the expected utility will be reduced. This will directly impact the reduction of retail prices. Yet the market demand will increase. Hence, retailer’s expected utility and social welfare increase.

Proposition 4: The risk aversion degree of retailer is inversely proportional to the proportion of government subsidies, retail price and retailer expected utility in the two-decision models of risk aversion only for retailer and also risk aversion for both manufacturer and retailer. Wholesale price, green degree, manufacturer’s expected utility and social welfare are proportional.

From the propositions described above, it can be found that in proposition 4 when the risk aversion of supply chain participants is higher, their own expected utility will decrease. Retailer can avoid risks by adjusting the retail prices. The higher the degree of risk aversion is, the lower the retail prices will be, and retail expected utility decrease accordingly. When the risk aversion degree of retailer is low, the bargaining power of retailer will increase. At this time, manufacturer needs a higher proportion of government subsidies. Thus, the wholesale price, greening degree and manufacturer’s expected utility will decrease correspondingly, which ultimately affects the reduction of social welfare.

## 5. Comparison and analysis of models

According to the Hesse matrix in the above model and all decision variables are positive, it can be concluded that: 16βη>30γ^2^, α>βcA_1_, B_1_, B_2_>0.

According to Table 1, the comparison among the optimal results of each variable of the four-decision models can be obtained as follows:

Corollary 1: Optimal proportion of government green subsidies: $\lambda_{2}^{*}<\lambda_{1}^{*}<\lambda_{4}^{*}<\lambda_{3}^{*}$;

Comparing with government green subsidy ratio, the MA model has the highest proportion of optimal government green subsidies, while the RA model has the lowest proportion. When the manufacturer is risk-averse and the retailer is not, the government will increase the proportion of green subsidies to encourage the manufacturer to increase green investment and to improve the greenness of the product. However, when retailer is risk-averse and manufacturer is not, the government’s optimal green subsidy ratio is the lowest. It can be seen that the more risk-averse manufacturer is, the bigger the proportion of green subsidies will be. While the lowest subsidy ratio provided by the government can also keep the product high greenness when retailers are risk-averse. Because the manufacturer will take the initiative to improve the product greenness to avoid market risks. The proofs are shown in Appendix A in S1 File.

Corollary 2: Optimal product greenness: when 2φ_m_<φ_r_, $e_{1}^{*}<e_{2}^{*}<e_{3}^{*}<e_{4}^{*}$; when 2φ_m_<φ_r_, $e_{1}^{*}<e_{3}^{*}<e_{2}^{*}<e_{4}^{*}$;

Comparing with product greenness, BA model has the highest greenness of products, while BN model has the lowest greenness of products. The greenness of products is the highest when all participants in the supply chain are risk averse, indicating that when both manufacturer and retailer consider risk aversion, and the market situation is relatively tense. Manufacturer will try its best to provide products with higher greenness to attract customers and increase sales. The proportion of government green subsidy in RA model is lower than that in BN model, yet the greenness in RA model is higher than that in BN, which indicates that retailer have risk aversion and can improve the greenness of products. In the meantime, in the model of RA and MA, when 2φ_m_<φ_r_, $e_{3}^{*}<e_{2}^{*}$, it shows that the manufacturer’s risk aversion has a greater impact on the products’ greenness than the retailer’s risk aversion. It can be found that the risk aversion characteristic of the retailer is conducive to increase the green effect of products and promoting the development of green supply chain. While the risk-averse manufacturer has negative effects on the development of green supply chain. The proofs are shown in Appendix B in S1 File.

Corollary 3: Optimal retail price of the product: when 2φ_m_>φ_r_, $p_{4}^{*}<p_{3}^{*}<p_{2}^{*}<p_{1}^{*};$ when 2φ_m_<φ_r_, $p_{4}^{*}<p_{2}^{*}<p_{3}^{*}<p_{1}^{*}$;

Comparing with retailer price, the retail price in BN model is the highest, whereas the retail price in BA is the lowest. When all participants in the supply chain consider risk aversion, retailer will increase its share by adjusting the retail prices to avoid risks. When 2φ_m_>φ_r_, $p_{2}^{*}>p_{3}^{*}$, first, it shows that the risk aversion of manufacturer is highly sensitive to the impact of retail prices. Second, in this case, the manufacturer does not consider the risk aversion, but the retailer does. So the manufacturer will set a higher wholesale price, and the retailer will also increase the retail price to ensure its own expected utility. But when 2φ_m_<φ_r_, manufacturer considers the risk aversion whereas retailer does not. In this case, when the wholesale price is low, retailer will also lower the retail price to attract customers. It can be seen that retailer reduces the retail price of products to attract consumers, to avoid risks and to achieve economies of scale. The proofs are shown in Appendix C in S1 File.

Corollary 4: Optimal social welfare: when 2φ_m_<φ_r_, ${\Pi_{sw}}_{1}^{*}<{\Pi_{sw}}_{2}^{*}<{\Pi_{sw}}_{3}^{*}<{\Pi_{sw}}_{4}^{*}$; when 2φ_m_>φ_r_, ${\Pi_{sw}}_{1}^{*}<{\Pi_{sw}}_{3}^{*}<{\Pi_{sw}}_{2}^{*}<{\Pi_{sw}}_{4}^{*}$.

Comparing with social welfare, the optimal social welfare is the highest in the BA mode. When both the manufacturer and the retailer consider risk aversion, the optimal green degree of the product is the highest, and the optimal expected utility of the retailer and the manufacturer is in the middle level. But in the BN model, the optimal social welfare is minimum. When 2φ_m_>φ_r_, manufacturer has low bargaining power. In order to make up for their negative impact on the green supply chain, the government subsidizes them to improve the greenness of their products, so ${\Pi_{sw}}_{2}^{*}>{\Pi_{sw}}_{3}^{*}$. The same as when 2φ_m_<φ_r_, ${\Pi_{sw}}_{2}^{*}<{\Pi_{sw}}_{3}^{*}$. It can be seen that the moderate risk aversion of supply chain participants can benefit the economy and sociality of the green supply chain. The proofs are shown in Appendix D in S1 File.

Corollary 5: Optimal wholesale price of products: when $32\beta \eta \varphi_{m}(\beta +\varphi_{r})C_{4}-\gamma^{2}(\beta +2\varphi_{r})(D_{1}+D_{2})>0,\omega_{3}^{*}<\omega_{4}^{*}<\omega_{1}^{*}<\omega_{2}^{*};$ when $32\beta \eta \varphi_{m}(\beta +\varphi_{r})C_{4}-\gamma^{2}(\beta +2\varphi_{r})(D_{1}+D_{2})<0,\omega_{3}^{*}<\omega_{1}^{*}<\omega_{4}^{*}<\omega_{2}^{*};$

Comparing with wholesale price, the optimal wholesale price is the highest in the RA model and the lowest in the MA model, which is consistent with the reality. Because the manufacturer has the highest bargaining power when the retailer is risk averse and the manufacturer is not. However, when the manufacturer is risk averse and the retailer is not, the manufacturer will adjust the wholesale price to avoid the risk. The higher the risk aversion of the manufacturer is, the lower its bargaining power will be. It can be seen that the more the manufacturer afraid of risks is, the more conservative the decision-making behavior is, which leads to the weakening of its bargaining power and the strengthening of the bargaining power of the corresponding retailer. The proofs are shown in Appendix E in S1 File.

Corollary 6: Manufacturer’s optimal expected utility: when$16\beta \eta C_{4}(2\beta^{2}\varphi_{m}-\varphi_{r}\beta^{2}-2\beta {\varphi_{r}}^{2}+4\varphi_{r}\beta \varphi_{m}+2\varphi_{m}{\varphi_{r}}^{2})-\gamma^{2}(\beta +{2\varphi }_{r})D_{3}>0,{U(\Pi_{m3})}^{*}<{U(\Pi_{m4})}^{*}<{U(\Pi_{m1})}^{*}<{U(\Pi_{m2})}^{*};$ when $16\beta \eta C_{4}(2\beta^{2}\varphi_{m}-\varphi_{r}\beta^{2}-2\beta {\varphi_{r}}^{2}+4\varphi_{r}\beta \varphi_{m}+2\varphi_{m}{\varphi_{r}}^{2})-\gamma^{2}(\beta +{2\varphi }_{r})D_{3}<0,{U(\Pi_{m3})}^{*}<{U(\Pi_{m1})}^{*}<{U(\Pi_{m4})}^{*}<{U(\Pi_{m2})}^{*}$.

Comparing with manufacturer’s expected utility, the manufacturer’s optimal expected utility is the highest in RA model and the lowest in MA model. When the manufacturer avoids risks, the manufacturer will reduce the greenness and the wholesale price to avoid risks, which the sales volume and the price will reduce at the same time, so thus the expected utility reduces. When retailer avoids risks, it will reduce the selling price to increase the market demand as well as to increase the sales share of manufacturer for the purpose of improving its expected utility. It can be seen that when the manufacturer is risk averse, its conservative decision based on risk considerations is good for the retailer. The proofs are shown in Appendix F in S1 File.

Corollary 7: Optimal expected utility for the retailer: when $256\beta^{2}\eta^{2}{C_{4}}^{2}D_{4}-\gamma^{2}D_{5}>0,{U(\Pi_{r2})}^{*}<{U(\Pi_{r1})}^{*}<{U(\Pi_{r4})}^{*}<{U(\Pi_{r3})}^{*}$, when $256\beta^{2}\eta^{2}{C_{4}}^{2}D_{4}-\gamma^{2}D_{5}<0,{U(\Pi_{r2})}^{*}<{U(\Pi_{r4})}^{*}<{U(\Pi_{r1})}^{*}<{U(\Pi_{r3})}^{*}$.

Comparing with retailer’s expected utility, the retailer’s optimal expected utility is the highest in MA model and the lowest in RA model. When the retailer considers risk aversion and the manufacturer does not, the manufacturer has strong bargaining power, and the retailer will adjust the retail price to avoid risks. Therefore, the retailer has the optimal expected utility in MA model and the lowest expected utility in RA model. Nevertheless, in MA model, the manufacturer adopts conservative decision-making behavior to lower the wholesale price of the product. Given this circumstance, the retailer can obtain higher expected utility without considering risk aversion. It can be seen that when the retailer is risk averse, its conservative decision based on risk considerations is good for the manufacturer. The proofs are shown in Appendix G in S1 File.

## 6. Numerical simulations

In order to verify the correctness of the above conclusions and models, we assume α = 200, β = 2, c = 20, γ = 1, η = 1, φ_m_ = 1, and φ_r_ = 1 by referring to Li et al. [51], Liu et al. [52] and combining it with the assumptions in the previous section of this paper.

The sensitivity of exogenous parameters and the influence of risk aversion on the optimal decision-making, utility, and society welfare of the manufacturer, retailer and government are analyzed by drawing with Python software.

(1) Sensitivity analysis of parameter β, γ, η on Π_sw_

**Fig 1:**
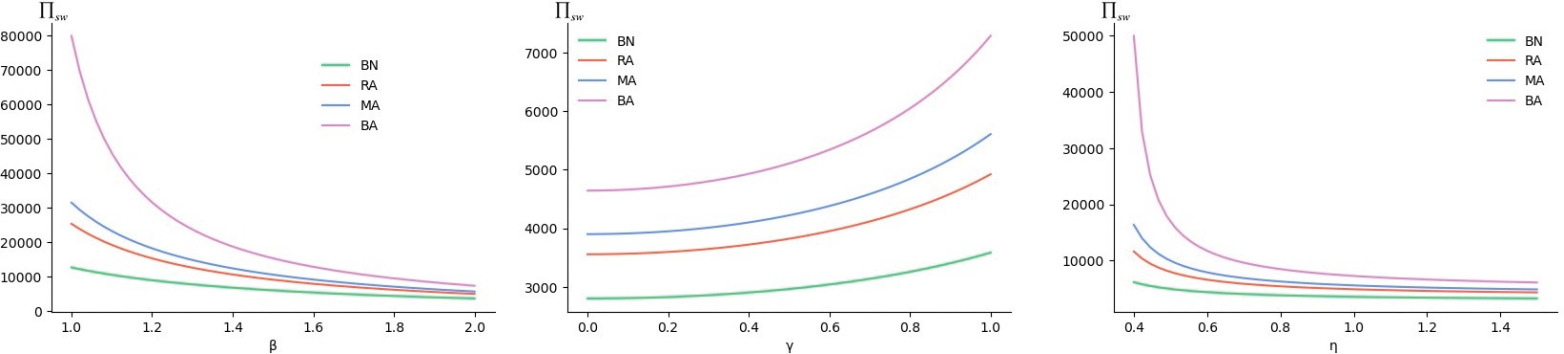
Sensitivity analysis of β, γ, η parameter on Π_sw_.

As can be seen from Fig 1A–1C, in four different decision models, β, η are inversely proportional to Π_sw_, while γ is positively proportional to Π_sw_; In other words, with the decrease of retail price sensitivity coefficient (β) and green cost invest coefficient (η) as well as the increase of consumers’ green preference coefficient (γ), social welfare (Π_sw_) increase. Consistent with proposition1, it also conforms to the actual situation. At the same time, the BA model has the maximum social welfare, and the BN model has the minimum social welfare under this assignment condition. It shows that the maximization of social welfare is the highest in these four models when both retailer and manufacturer consider risk aversion. When both retailer and manufacturer do not consider risk aversion, the maximization of social welfare is the lowest in these four models. Thus, in the long run, the development of green supply chain still depends on the improvement of consumer awareness and enterprise green technology.

(2) The influence of risk aversion on decision variables

**Fig 2:**
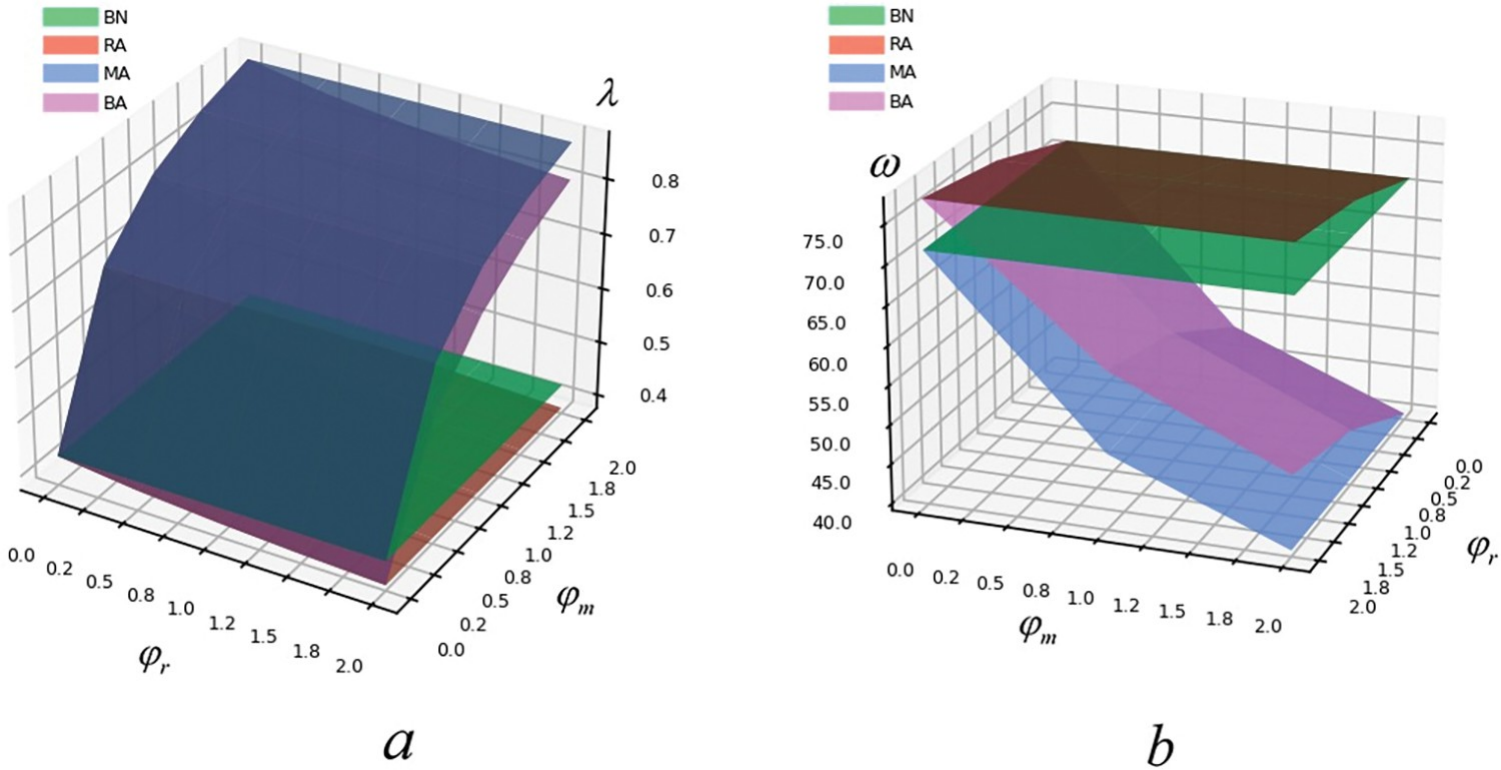
The influence of risk aversion on λ and ω.

From Fig 2A and 2B, it is known that the parameters of BN model are not affected by risk aversion. From Fig 2A, it points out that no matter how risk averse retailer and manufacturer are, $\lambda_{2}^{*}<\lambda_{1}^{*}<\lambda_{4}^{*}<\lambda_{3}^{*}$, which is consistent with the previous proposition. The MA model and BA model are proportional to the parameter λ. BA model is inversely proportional to RA model, but the influence is limited. It shows that only when the manufacturer considers risk aversion, the manufacturer will take measures to reduce the product greenness to avoid risks. Therefore, the government increases the subsidy ratio to the manufacturer to ensure the product greenness. Government subsidy policy can effectively reduce the negative impact on the supply chain in terms of manufacturer’s consideration of risk aversion, which has a positive effect on the development of green supply chain. Thus, it means that the more risk averse manufacturers are, the stronger the need for government subsidies is.

From Fig 2B, it shows that with the increase of manufacturer’s risk aversion, the wholesale prices (ω) of MA model and BA model decrease; With the increase of retailer risk aversion, the wholesale prices (ω) of RA model and BA model increase. Yet, regardless of the increase or decrease of φ_m_ and φ_r_, the wholesale price is always the highest in RA model and the lowest in MA model. The manufacturer, as a leader, has strong bargaining power. When the manufacturer considers risk aversion, and the retailer does not, the manufacturer will make decisions prudently, which will reduce its bargaining power. In the meantime, the higher the manufacturer’s risk aversion is, the lower its bargaining power will be. Thus, the wholesale price will be lower. On the contrary, when the retailer considers risk aversion, yet the manufacturer does not, the manufacturer will have stronger bargaining power, which increases the wholesale price. Therefore, it means that the bargaining power of manufacturer decreases with the increase of risk aversion.

**Fig 3:**
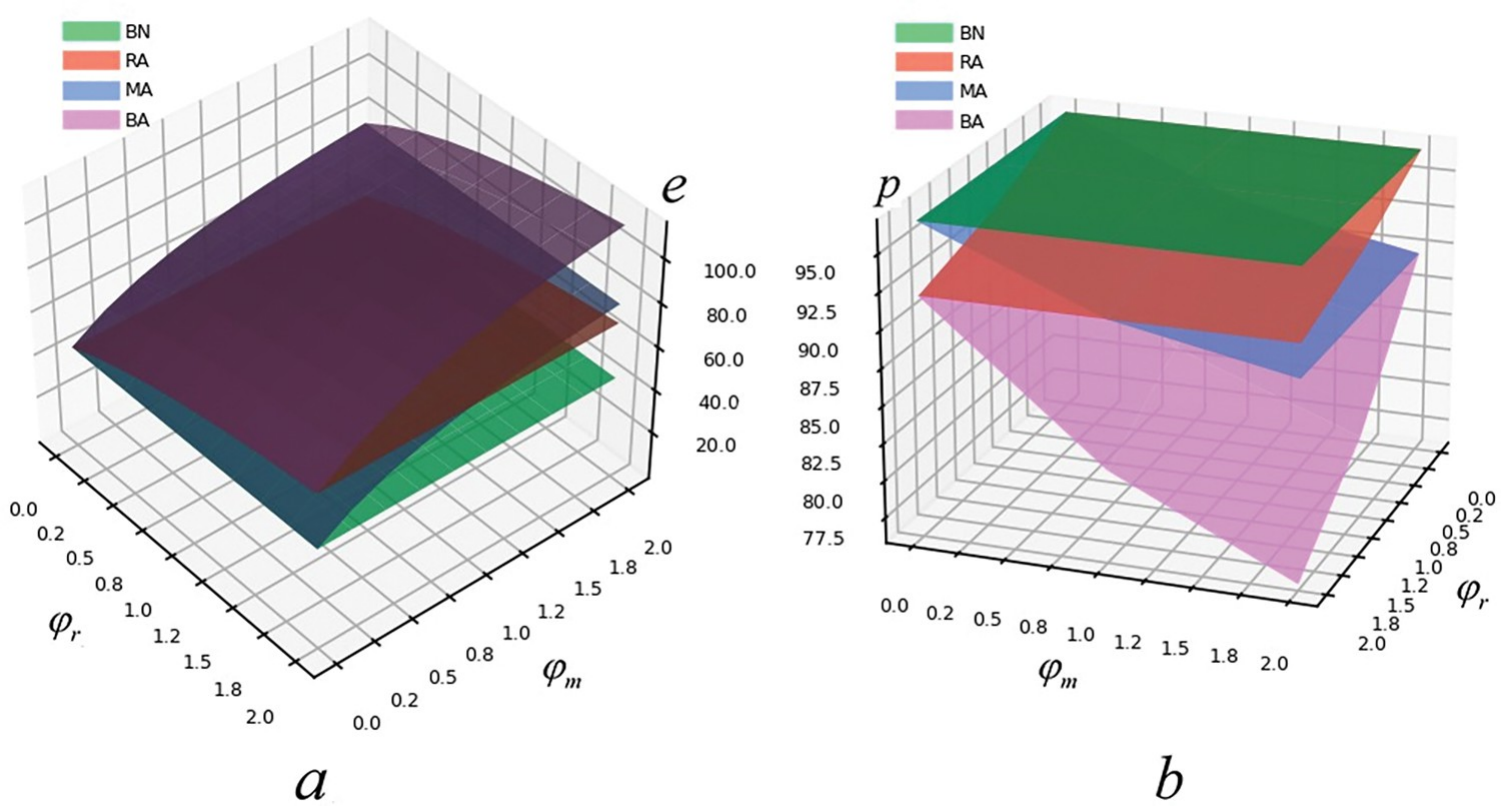
The influence of risk aversion on e and p.

It can be found in Fig 3A and 3B that excepted BN model manufacturer’s risk aversion and retailer’s risk aversion are directly proportional to green degree (e) and inversely proportional to retail price (p). In BA model, the green degree is the highest and the retail price is the lowest; In BN model, the green degree is the lowest and the retail price is the highest. It is revealed that when both retailer and manufacturer consider risk aversion, they will adopt conservative strategies, which leads to the reduction of retail price. However, due to the government’s sufficient green subsidies, the green degree of products will not decrease but increase so that consumers can purchase good products with relative lower price. Thus, it means that the bargaining power of retailer decreases with the increase of risk aversion. Moreover, manufacturers’ risk aversion has a damaging effect on product greenness, and retailers’ risk aversion has an enhancing effect on product greenness.

**Fig 4:**
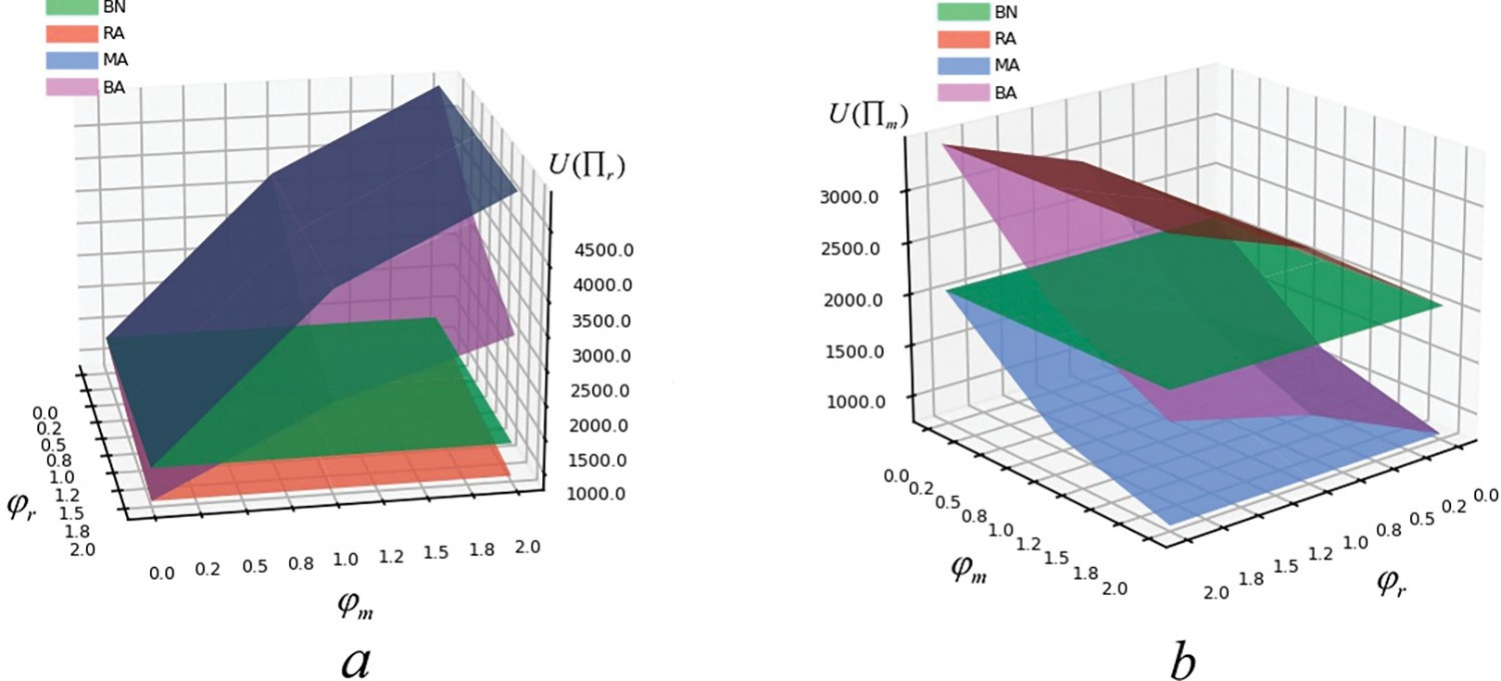
The influence of risk aversion on U(Π_m_) and U(Π_r_).

Fig 4A and 4B presents that in MA model and BA model, manufacturer’s expected utility decreases with the increase of manufacturer’s risk aversion, while retailer’s expected utility increases with the increase of manufacturer’s risk aversion. In RA and BA models, manufacturer’s expected utility is positively correlated with retailer’s risk aversion, and retailer’s expected utility is negatively correlated with retailer’s risk aversion. Meanwhile, the expected utility of manufacturer is the highest and the expected utility of retailer is the lowest in RA model. In MA model, retailer’s expected utility is the highest and manufacturer’s expected utility is the least. The reason is that in this kind of game, when the retailer considers risk aversion and the manufacturer does not, the manufacturer has strong bargaining power. In view of this, the wholesale price can be put up and the manufacturer’s expected utility also increases. Moreover, the higher the retailer’s risk aversion is, the greater the manufacturer’s expected utility will be. RA model is in line with this. When there is a strong player in the supply chain, the conservative strategy adopted by the weaker one cannot get objective expected utility. On the contrary, when both the manufacture and the retailer consider risk aversion or do not consider risk aversion, there will be equal strength. Therefore, both players of the supply chain need to reasonably evaluate other’s risk aversion and make reasonable decisions accordingly. Hence, it means that the risk aversion of manufacturer and retailer harms their expected utility.

**Fig 5:**
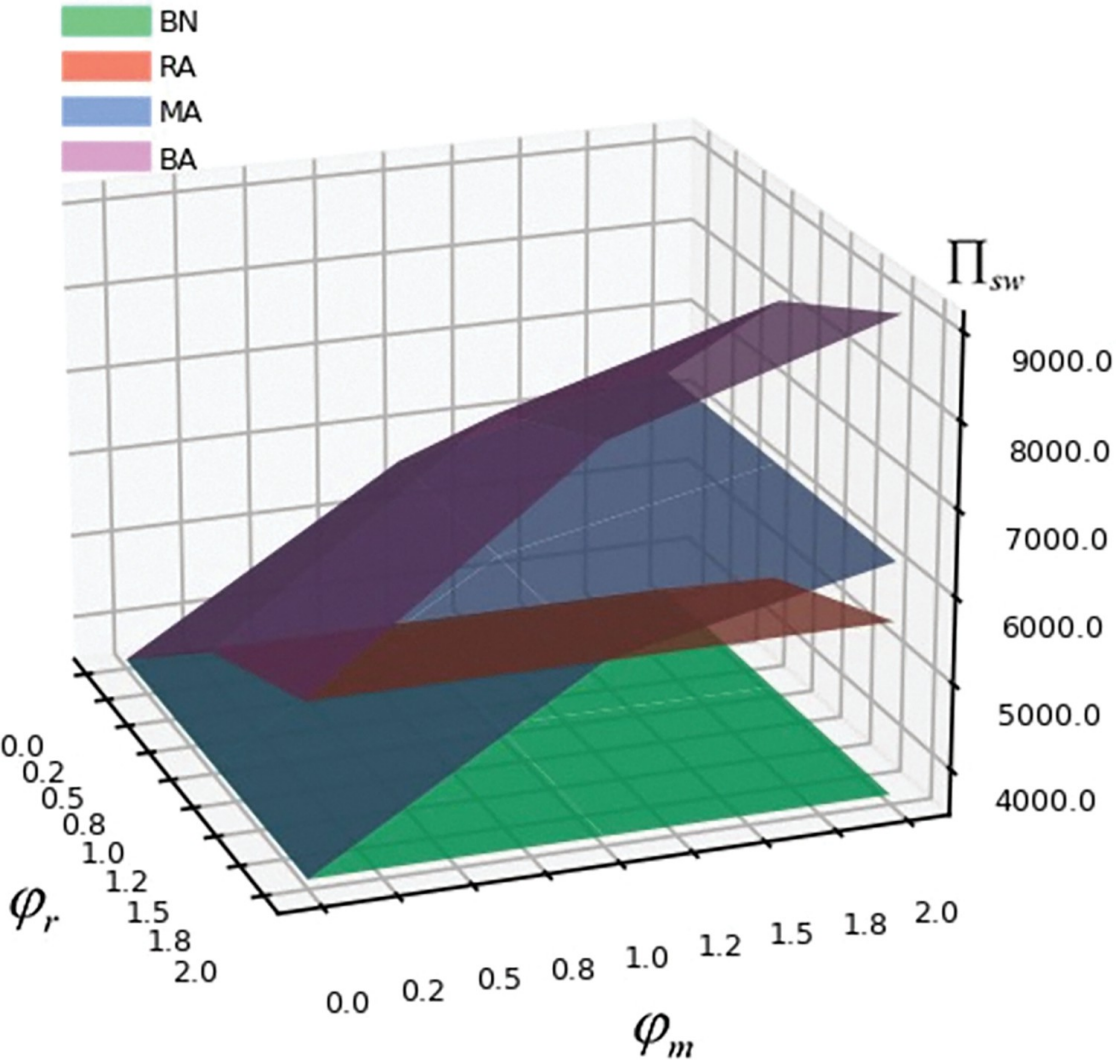
The influence of risk aversion on Π_sw_.

Fig 5 illustrates that the risk aversion of manufacturer and retailer enhances social welfare. BA model has the highest social welfare, and BN model has the lowest social welfare. This attributes to the risk aversion consideration from manufacturer and retailer. To guarantee the green degree of products, the government will increase the proportion of subsidies to manufacturer to improve social welfare. When manufacturer and retailer do not consider risk aversion, manufacturer will improve the green degree of products to attract more customers. However, the proportion of government subsidies will be reduced, so will the social welfare.

## 7. Conclusion and enlightenment

Considering the green preference of consumers, the risk aversion of manufacturer and retailer, this paper constructs four different game models of three-level green supply chain composed of government, one manufacturer and one retailer, finds out the corresponding equilibrium solutions and analyzes the sensitivity of each parameter. By means of theoretical analysis and numerical simulations, whether manufacturer and retailer should consider the impact of risk aversion on various factors has been analyzed.

The research reveals that:

(1) When supply chain participants consider risk aversion at the same time or do not consider risk aversion at the same time, there will be a situation of equal strength. Therefore, enterprises should reasonably control their own risk aversion and evaluate the risk aversion of upstream and downstream enterprises rationally so that they can make decisions correspondingly.

(2) When the manufacturer considers the risk aversion, the government will give the manufacturer green subsidies. The higher the risk aversion is, the higher the proportion of government subsidies will be. When both retailer and manufacturer consider risk aversion, they will adopt conservative strategies, which will lead to the reduction of retail prices. However, in order to ensure the green degree of products in the green supply chain, the government will give sufficient green degree subsidies so that the green degree of products does not decrease but increases. Meanwhile, consumers can consume products with good quality and relative lower price. The government subsidy to manufacturer is conducive to the development of green supply chain. Additionally, the government subsidy policy can effectively limit the negative impact of manufacturer’s risk aversion on green supply chain.

(3) For supply chain participants, in order to survive in the fierce competition market, green production enterprises should strive to improve their green production level and innovation ability as well as to enhance their core competitiveness. They should also incorporate green innovation into the enterprise development strategic plan, continuously carry out green innovation research and develop green technology innovation. In addition, enterprises should take effective measures to control their own risk aversion behavior when making decisions. On the one hand, enterprise shall pay attention to market dynamics in time, actively use historical data and information means to predict product demand, take prior measures to control enterprise risks and strategize and make reasonable decisions. On the other hand, the enterprise’s risk information system shall be improved, the risks shall be identified and correctly evaluated in time and the results must be given actively. Enterprises can also establish a rapid response mechanism based on information systems, set up a rapid response index system and its range and critical line, timely capture risk signs, and remind relevant decision makers and managers to take preventive and to solve measures in advance.

(4) For the government, relevant departments should monitor market behavior in real time. Good market order is the guarantee of green production and green innovation. It can not only improve economic efficiency but also better allocate existing resources. What’s more, the government should comprehensively consider the interests of all parties and green benefits, and improve the policies related to green technology subsidies. Therefore, the government should reasonably set the level of financial subsidies according to the actual situation to promote enterprises to carry out sustainable innovation. The government should guide enterprises to innovate constantly and inject new impetus into the realization of sustainable development.

This paper provides a theoretical basis for decision-makers to take effective strategies in three-level green supply chain with green investment subsidy. However, the impact of only one government subsidy mode in different models is discussed here. In the future, the manufacturer and retailer taking the impact of risk aversion on supply chain into consideration with different government subsidy modes could be explored.

## Supporting information

S1 File Contains Appendix A–G. (DOCX)
