# Supplementary material for: Supply chain decision based on green investment subsidy and risk aversion
Source: PLoS One. 2023 Nov 6;18(11):e0293924. doi: 10.1371/journal.pone.0293924 (PMC10627464; doi:10.1371/journal.pone.0293924)
Supplement: S1 File — (DOCX) [file pone.0293924.s001.docx]

Appendix A. Compare with the optimal proportion of government green subsidies

$\lambda_{4}^{*}-\lambda_{3}^{*}=\frac{-4\beta\varphi_{r}\left( 4{\varphi_{r}}^{2}\left( 32{\varphi_{r}}^{3} + 48\beta{\varphi_{r}}^{2} + 19\beta^{2}\varphi_{m} + \beta^{3} \right)+\beta\left( \beta^{3}\left( \beta+ 36\varphi_{m}+ 4\varphi_{r} \right)+2\varphi_{m}\left( 64\beta^{2}\varphi_{m} + 55\varphi_{r}\beta^{2}+ 64\beta{\varphi_{m}}^{2} + 160\varphi_{r}\beta\varphi_{m} + 128\varphi_{r}{\varphi_{m}}^{2} \right) \right) \right)}{\left( 48{\varphi_{m}}^{2} + 40\beta\varphi_{m} + 7\beta^{2} \right)\left( 4{\varphi_{r}}^{2}\left( 13\beta^{3}+ 44\beta^{2}\varphi_{m}+ 6\varphi_{r}\beta^{2}+ 28\beta{\varphi_{m}}^{2} + 16\varphi_{r}\beta\varphi_{m} + 8\varphi_{r}{\varphi_{m}}^{2} \right)+\beta^{2}\left( 7\beta^{3}+ 40\beta^{2}\varphi_{m} + 34\varphi_{r}\beta^{2} + 48\beta{\varphi_{m}}^{2} + 152\varphi_{r}\beta\varphi_{m} + 128\varphi_{r}{\varphi_{m}}^{2} \right) \right)}<0$，i.e. $\lambda_{4}^{*}<\lambda_{3}^{*}$；

$\lambda_{1}^{*}-\lambda_{4}^{*}=-\frac{4\left( \beta^{2}\left( 26\beta^{2}\varphi_{m}+\varphi_{r}\beta\left( 96\varphi_{m}-\beta\right)+ 48\beta{\varphi_{m}}^{2} + 128\varphi_{r}{\varphi_{m}}^{2} \right)+2{\varphi_{r}}^{2}\left( \beta^{2}\left( {53\varphi}_{m}-2\beta\right)+{2\varphi}_{r}\beta\left( 9\varphi_{m}-\beta\right)+ 56\beta{\varphi_{m}}^{2}+ 16\varphi_{r}{\varphi_{m}}^{2} \right) \right)}{7\left( 4{\varphi_{r}}^{2}\left( 13\beta^{3}+ 44\beta^{2}\varphi_{m}+ 6\varphi_{r}\beta^{2}+ 28\beta{\varphi_{m}}^{2} + 16\varphi_{r}\beta\varphi_{m} + 8\varphi_{r}{\varphi_{m}}^{2} \right)+\beta^{2}\left( 7\beta^{3}+ 40\beta^{2}\varphi_{m} + 34\varphi_{r}\beta^{2} + 48\beta{\varphi_{r}}^{2} + 152\varphi_{r}\beta\varphi_{m} + 128\varphi_{r}{\varphi_{m}}^{2} \right) \right)}<0$, because $9\varphi_{m}-\beta>0$, ${53\varphi}_{m}-2\beta>0$, $\lambda_{1}^{*}-\lambda_{4}^{*}<0$, i.e. $\lambda_{1}^{*}<\lambda_{4}^{*}$;

$\lambda_{2}^{*}-\lambda_{1}^{*}=-\frac{4\varphi_{r}}{7\left( 7\beta+ 6\varphi_{r} \right)}<0$，即$\lambda_{2}^{*}<\lambda_{1}^{*}$；

To sum up, we can get $\lambda_{2}^{*}<\lambda_{1}^{*}<\lambda_{4}^{*}<\lambda_{3}^{*}$.

Appendix B. Compare with the optimal product greenness

$e_{4}^{*}-e_{2}^{*}=\frac{64\beta\gamma\eta\varphi_{m}\left( \beta+\varphi_{r} \right)^{3}\left( \beta+2\varphi_{r} \right)\left( \alpha-\beta c \right)\left( 3\beta^{3}+8\beta^{2}\varphi_{r}+5\varphi_{m}\beta^{2}+4\beta{\varphi_{r}}^{2}+7\varphi_{r}\beta\varphi_{m}+2{\varphi_{r}}^{2}\varphi_{m} \right)}{A_{1}\left( 16\beta\eta{C_{4}}^{2}-\gamma^{2}C_{5}C_{6}\left( \beta+\varphi_{r} \right) \right)}$，Each decision variable is positive, so$16\beta\eta\left( \beta+2\varphi_{m} \right)^{2}-\gamma^{2}\left( 7\beta+12\varphi_{m} \right)\left( \beta+4\varphi_{m} \right)>0$，$16\beta\eta{C_{4}}^{2}-\gamma^{2}C_{5}C_{6}\left( \beta+\varphi_{r} \right)>0$，i.e. $e_{4}^{*}>e_{2}^{*}$.

$e_{4}^{*}-e_{3}^{*}=\frac{16\beta\gamma\eta\varphi_{r}\left( \alpha-\beta c \right)\left( 4{\varphi_{r}}^{2}\left( 11\beta^{5} + 63\beta^{4}\varphi_{m} + 5\varphi_{r}\beta^{4} + 156\beta^{3}{\varphi_{m}}^{2}+ 26\varphi_{r}\beta^{3}\varphi_{m} + 176\beta^{2}{\varphi_{m}}^{3} + 57\varphi_{r}\beta^{2}{\varphi_{m}}^{2} + 64\beta{\varphi_{m}}^{4} + 56\varphi_{r}\beta{\varphi_{m}}^{3} + 16\varphi_{r}{\varphi_{m}}^{4} \right) \right)}{B_{1}\left( 16\beta\eta{C_{4}}^{2}-\gamma^{2}\left( \beta+2\varphi_{r} \right)C_{5}C_{6} \right)}+\frac{16\beta\gamma\eta\varphi_{r}\left( \alpha-\beta c \right)\left( \beta^{2}\left( 6\beta^{5} + 44\beta^{4}\varphi_{m} + 29\varphi_{r}\beta^{4} + 144\beta^{3}{\varphi_{m}}^{2} + 188\varphi_{r}\beta^{3}\varphi_{m} + 224\beta^{2}{\varphi_{m}}^{3} + 536\varphi_{r}\beta^{2}{\varphi_{m}}^{2} + 128\beta{\varphi_{m}}^{4} + 704\varphi_{r}\beta{\varphi_{m}}^{3} + 320\varphi_{r}{\varphi_{m}}^{4} \right) \right)}{B_{1}\left( 16\beta\eta{C_{4}}^{2}-\gamma^{2}\left( \beta+2\varphi_{r} \right)C_{5}C_{6} \right)}$；Refer to table 1, because each decision variable is positive，$e_{4}^{*}-e_{3}^{*}>0$，i.e. $e_{4}^{*}>e_{3}^{*}$；

$e_{2}^{*}-e_{1}^{*}=\frac{16\beta\gamma\eta\varphi_{r}\left( \alpha-\beta c \right)\left( 6\beta+5\varphi_{r} \right)}{A_{1}\left( 16\beta\eta-7\gamma^{2} \right)}$，$\alpha-\beta c>0$；$A_{1}>0$；$16\beta\eta-7\gamma^{2}>0$，so $e_{2}^{*}-e_{1}^{*}>0$，i.e. $e_{2}^{*}>e_{1}^{*}$；

$e_{3}^{*}-e_{1}^{*}=\frac{64\beta\gamma\eta\varphi_{m}\left( \alpha-\beta c \right)\left( 3\beta+5\varphi_{m} \right)}{B_{1}\left( 16\beta\eta-7\gamma^{2} \right)}>0$，i.e. $e_{3}^{*}>e_{1}^{*}$；

$e_{3}^{*}-e_{2}^{*}=\frac{16\beta^{2}\eta\gamma\left( \alpha-\beta c \right)\left( 2\varphi_{m}-\varphi_{r} \right)\left( 10\beta\varphi_{m}+5\beta\varphi_{r}+8\varphi_{m}\varphi_{r}+6\beta^{2} \right)}{A_{1}B_{1}}$；when $2\varphi_{m}-\varphi_{r}>0$, $e_{3}^{*}-e_{2}^{*}>0$, i.e. $e_{3}^{*}>e_{2}^{*}$; when $2\varphi_{m}-\varphi_{r}<0, e_{3}^{*}-e_{2}^{*}<0$, i.e. $e_{3}^{*}<e_{2}^{*}$.

Appendix C. Compare with the optimal retail price of the product

$p_{1}^{*}-p_{2}^{*}=\frac{4\eta\varphi_{r}\left( \alpha-\beta c \right)\left( 16\eta\beta\left( \beta+\varphi_{r} \right)-\gamma^{2}\left( 25\beta+22\varphi_{r} \right) \right)}{A_{1}\left( 16\beta\eta-7\gamma^{2} \right)}$，because $16\eta\beta>{30\gamma}^{2}$, $16\eta\beta\left( \beta+\varphi_{r} \right)-\gamma^{2}\left( 25\beta+22\varphi_{r} \right)>0$，$p_{1}^{*}-p_{2}^{*}>0$，$p_{1}^{*}>p_{2}^{*}$;

Similarly,

$p_{1}^{*}-p_{3}^{*}=\frac{8\eta\varphi_{m}\left( \alpha-\beta c \right)\left( 16\eta\beta\left( \beta+2\varphi_{m} \right)-\gamma^{2}\left( 25\beta+44\varphi_{m} \right) \right)}{B_{1}\left( 16\beta\eta-7\gamma^{2} \right)}>0$;

$p_{2}^{*}-p_{4}^{*}=\frac{8\eta\varphi_{m}\left( \alpha-\beta c \right)\left( \beta+\varphi_{r} \right)^{2}\left( \beta+2\varphi_{r} \right)\left( 16\beta\eta\left( \beta+\varphi_{r} \right)C_{4}-\gamma^{2}\left( 4{\varphi_{r}}^{2}\left( 26\beta\varphi_{m} + 10\beta\varphi_{r} + 8\varphi_{m}\varphi_{r}+ 27\beta^{2} \right)+\beta^{2}\left( 44\beta\varphi_{m} + 94\beta\varphi_{r} + 116\varphi_{m}\varphi_{r} + 25\beta^{2} \right) \right) \right)}{A_{1}\left( 16\beta\eta{C_{4}}^{2}-\gamma^{2}C_{5}C_{6}\left( \beta+\varphi_{r} \right) \right)}>0$;

$p_{3}^{*}-p_{4}^{*}=\frac{4\eta\varphi_{r}\left( \alpha-\beta c \right)\left( \beta^{3}+4\beta^{2}\varphi_{m}+ 2\varphi_{r}\beta^{2} + 8\beta{\varphi_{m}}^{2} + 6\varphi_{r}\beta\varphi_{m} + 8\varphi_{r}{\varphi_{m}}^{2} \right)\left( 16\beta\eta\left( \beta+2\varphi_{m} \right)C_{4}-\gamma^{2}\left( 4{\varphi_{r}}^{2}\left( 16{\varphi_{m}}^{2} + 28\beta\varphi_{m} + 11\beta^{2} \right)+\beta\left( 25\beta^{3} + 88\beta^{2}\varphi_{m} + 72\varphi_{r}\beta^{2} + 80\beta{\varphi_{m}}^{2} + 204\varphi_{r}\beta\varphi_{m} + 144\varphi_{r}{\varphi_{m}}^{2} \right) \right) \right)}{B_{1}\left( 16\beta\eta{C_{4}}^{2}-\gamma^{2}C_{5}C_{6}\left( \beta+\varphi_{r} \right) \right)}>0$;

$p_{2}^{*}-p_{3}^{*}=\frac{4\beta\eta\left( \alpha-\beta c \right)\left( 2\varphi_{m}-\varphi_{r} \right)\left( 16\beta\eta\left( \beta+\varphi_{r} \right)\left( \beta+2\varphi_{m} \right)-\gamma^{2}\left( 44\beta\varphi_{m} + 22\beta\varphi_{r} + 40\varphi_{m}\varphi_{r} + 25\beta^{2} \right) \right)}{A_{1}B_{1}}$, $16\eta\beta>{30\gamma}^{2}$, so $16\beta\eta\left( \beta+\varphi_{r} \right)\left( \beta+2\varphi_{m} \right)-\gamma^{2}\left( 44\beta\varphi_{m} + 22\beta\varphi_{r} + 40\varphi_{m}\varphi_{r} + 25\beta^{2} \right)>0$, when $2\varphi_{m}-\varphi_{r}>0$, $p_{2}^{*}-p_{3}^{*}>0$, i.e. $p_{2}^{*}>p_{3}^{*}$; when $2\varphi_{m}-\varphi_{r}<0$, $p_{2}^{*}-p_{3}^{*}<0$, i.e. $p_{2}^{*}<p_{3}^{*}$.

Appendix D. Compare with the optimal social welfare

${\Pi_{\mathrm{sw}}}_{4}^{*}-{\Pi_{\mathrm{sw}}}_{3}^{*}=\frac{32\beta\eta^{2}{\varphi_{r}}^{3}\left( \alpha-\beta c \right)^{2}\left( 11\beta^{5} + 63\beta^{4}\varphi_{m}+ 5\varphi_{r}\beta^{4} + 156\beta^{3}{\varphi_{m}}^{2} + 26\varphi_{r}\beta^{3}\varphi_{m} + 176\beta^{2}{\varphi_{m}}^{3} + 57\varphi_{r}\beta^{2}{\varphi_{m}}^{2}+ 64\beta{\varphi_{m}}^{4} + 56\varphi_{r}\beta{\varphi_{m}}^{3}+ 16\varphi_{r}{\varphi_{m}}^{4} \right)}{4B_{1}\left( 16\beta\eta{C_{4}}^{2}-\gamma^{2}C_{5}C_{6}\left( \beta+\varphi_{r} \right) \right)}$

$+\frac{8\beta^{3}\eta^{2}\varphi_{r}\left( \alpha-\beta c \right)^{2}\left( 6\beta^{5} + 44\beta^{4}\varphi_{m} + 29\varphi_{r}\beta^{4} + 144\beta^{3}{\varphi_{m}}^{2} + 188\varphi_{r}\beta^{3}\varphi_{m} + 224\beta^{2}{\varphi_{m}}^{3} + 536\varphi_{r}\beta^{2}{\varphi_{m}}^{2}+ 128\beta{\varphi_{m}}^{4} + 704\varphi_{r}\beta{\varphi_{m}}^{3} + 320\varphi_{r}{\varphi_{m}}^{4} \right)}{4B_{1}\left( 16\beta\eta{C_{4}}^{2}-\gamma^{2}C_{5}C_{6}\left( \beta+\varphi_{r} \right) \right)}$, because the decision variables are positive, $16\beta\eta{C_{4}}^{2}-\gamma^{2}C_{5}C_{6}\left( \beta+\varphi_{r} \right)>0,$ ${\Pi_{\mathrm{sw}}}_{4}^{*}-{\Pi_{\mathrm{sw}}}_{3}^{*}>0,{\Pi_{\mathrm{sw}}}_{4}^{*}>{\Pi_{\mathrm{sw}}}_{3}^{*}$;

${\Pi_{\mathrm{sw}}}_{4}^{*}-{\Pi_{\mathrm{sw}}}_{2}^{*}=\frac{32\beta\eta^{2}\varphi_{m}\left( \beta+\varphi_{r} \right)^{3}\left( \beta+2\varphi_{r} \right)\left( \alpha-\beta c \right)^{2}\left( 3\beta^{3} + 8\beta^{2}\varphi_{r}+ 5\varphi_{m}\beta^{2}+ 4\beta{\varphi_{r}}^{2} + 7\varphi_{m}\beta\varphi_{r}+ 2\varphi_{m}{\varphi_{r}}^{2} \right)}{4A_{1}\left( 16\beta\eta{C_{4}}^{2}-\gamma^{2}C_{5}C_{6}\left( \beta+\varphi_{r} \right) \right)}>0$, i.e. ${\Pi_{\mathrm{sw}}}_{4}^{*}>{\Pi_{\mathrm{sw}}}_{2}^{*}$;

${\Pi_{\mathrm{sw}}}_{2}^{*}-{\Pi_{\mathrm{sw}}}_{1}^{*}=\frac{8\beta\eta^{2}\varphi_{r}\left( \alpha-\beta c \right)^{2}\left( 6\beta+ 5\varphi_{r} \right)}{{4A}_{1}\left( 16\beta\eta-7\gamma^{2} \right)}$, because $16\beta\eta-7\gamma^{2}>0$, ${\Pi_{\mathrm{sw}}}_{2}^{*}-{\Pi_{\mathrm{sw}}}_{1}^{*}>0$, i.e. ${\Pi_{\mathrm{sw}}}_{2}^{*}>{\Pi_{\mathrm{sw}}}_{1}^{*}$;

${\Pi_{\mathrm{sw}}}_{3}^{*}-{\Pi_{\mathrm{sw}}}_{1}^{*}=\frac{32\beta\eta^{2}\varphi_{m}\left( \alpha-\beta c \right)^{2}\left( 3\beta+ 5\varphi_{m} \right)}{4B_{1}\left( 16\beta\eta-7\gamma^{2} \right)}>0$, i.e. ${\Pi_{\mathrm{sw}}}_{3}^{*}>{\Pi_{\mathrm{sw}}}_{1}^{*}$;

${\Pi_{\mathrm{sw}}}_{2}^{*}-{\Pi_{\mathrm{sw}}}_{3}^{*}=-\frac{8\beta^{2}\eta^{2}\left( \alpha-\beta c \right)^{2}\left( 2\varphi_{m}-\varphi_{r} \right)}{4A_{1}B_{1}}$, when $2\varphi_{m}-\varphi_{r}>0$, ${\Pi_{\mathrm{sw}}}_{2}^{*}-{\Pi_{\mathrm{sw}}}_{3}^{*}>0$, i.e. ${\Pi_{\mathrm{sw}}}_{2}^{*}>{\Pi_{\mathrm{sw}}}_{3}^{*}$; when $2\varphi_{m}-\varphi_{r}<0$, ${\Pi_{\mathrm{sw}}}_{2}^{*}-{\Pi_{\mathrm{sw}}}_{3}^{*}<0$, i.e. ${\Pi_{\mathrm{sw}}}_{2}^{*}<{\Pi_{\mathrm{sw}}}_{3}^{*}$.

Appendix E. Compare with the optimal wholesale price of products

$\omega_{1}^{*}-\omega_{2}^{*}=-\frac{8\eta\gamma^{2}\varphi_{r}\left( \alpha-\beta c \right)\left( 6\beta+5\varphi_{r} \right)}{A_{1}\left( 16\beta\eta-7\gamma^{2} \right)}<0$, i.e. $\omega_{1}^{*}<\omega_{2}^{*}$;

$\omega_{1}^{*}-\omega_{3}^{*}=\frac{16\eta\varphi_{m}\left( \alpha-\beta c \right)\left[ 16\beta\eta\left( \beta+2\varphi_{m} \right)-\gamma^{2}\left( 13\beta+24\varphi_{m} \right) \right]}{B_{1}\left( 16\beta\eta-7\gamma^{2} \right)}$,$8\beta\eta\left( \beta+2\varphi_{m} \right)-\gamma^{2}\left( 7\beta+12\varphi_{m} \right)>0$, i.e.$16\beta\eta\left( \beta+2\varphi_{m} \right)-\gamma^{2}\left( 14\beta+24\varphi_{m} \right)>0$, $16\beta\eta\left( \beta+2\varphi_{m} \right)-\gamma^{2}\left( 13\beta+24\varphi_{m} \right)>0$, so $\omega_{3}^{*}<\omega_{1}^{*}$;

$\omega_{4}^{*}-\omega_{3}^{*}=\frac{8\beta\eta\varphi_{r}\left( \alpha-\beta c \right)\left( 32\beta\eta\varphi_{m}\left( \beta+\varphi_{r} \right)\left( \beta+2\varphi_{m} \right)C_{4}-\gamma^{2}\left( \beta+2\varphi_{r} \right)\left( 2{\varphi_{r}}^{2}\left( 16{\varphi_{m}}^{3}+8\beta{\varphi_{m}}^{2}- 9\beta^{2}\varphi_{m}- 5\beta^{3} \right)+\beta\left( 32\beta{\varphi_{m}}^{3}+ 16\varphi_{r}\beta{\varphi_{m}}^{2}+ 64\varphi_{r}{\varphi_{m}}^{3}-6\beta^{4}- 18\beta^{3}\varphi_{m}- 17\varphi_{r}\beta^{3}- 38\varphi_{r}\beta^{2}\varphi_{m} \right) \right) \right)}{B_{1}\left( 16\beta\eta{C_{4}}^{2}-\gamma^{2}C_{5}C_{6}\left( \beta+\varphi_{r} \right) \right)}$，because$16\eta\beta>{30\gamma}^{2}$,$32\beta\eta\varphi_{m}\left( \beta+\varphi_{r} \right)\left( \beta+2\varphi_{m} \right)C_{4}-\gamma^{2}\left( \beta+2\varphi_{r} \right)\left( 2{\varphi_{r}}^{2}\left( 16{\varphi_{m}}^{3}+8\beta{\varphi_{m}}^{2}- 9\beta^{2}\varphi_{m}- 5\beta^{3} \right)+\beta\left( 32\beta{\varphi_{m}}^{3}+ 16\varphi_{r}\beta{\varphi_{m}}^{2}+ 64\varphi_{r}{\varphi_{m}}^{3}-6\beta^{4}- 18\beta^{3}\varphi_{m}- 17\varphi_{r}\beta^{3}- 38\varphi_{r}\beta^{2}\varphi_{m} \right) \right)>0$，$\omega_{4}^{*}-\omega_{3}^{*}$, i.e., $\omega_{4}^{*}>\omega_{3}^{*}$;

Similarly,

$\omega_{4}^{*}-\omega_{2}^{*}=-\frac{16\eta\varphi_{m}\left( \alpha-\beta c \right)\left( \beta+\varphi_{r} \right)^{3}\left( 16\beta\eta\left( \beta+\varphi_{r} \right)C_{4}-\gamma^{2}\left( \beta+2\varphi_{r} \right)\left( 13\beta^{3}+ 36\beta^{2}\varphi_{r}+ 24\beta^{2}\varphi_{m} + 20\beta{\varphi_{r}}^{2} + 40\varphi_{m}\varphi_{r}\beta+ 16\varphi_{m}{\varphi_{r}}^{2} \right) \right)}{A_{1}\left( 16\beta\eta{C_{4}}^{2}-\gamma^{2}C_{5}C_{6}\left( \beta+\varphi_{r} \right) \right)}>0$

$\omega_{4}^{*}-\omega_{1}^{*}=\frac{-8\eta\left( \alpha-\beta c \right)\left( 32\beta\eta\varphi_{m}\left( \beta+\varphi_{r} \right)C_{4}-\gamma^{2}\left( \beta+2\varphi_{r} \right)\left( D_{1}+D_{2} \right) \right)}{\left( 16\beta\eta-7\gamma^{2} \right)\left( 16\beta\eta{C_{4}}^{2}-\gamma^{2}C_{5}C_{6}\left( \beta+\varphi_{r} \right) \right)}$，when $32\beta\eta\varphi_{m}\left( \beta+\varphi_{r} \right)C_{4}-\gamma^{2}\left( \beta+2\varphi_{r} \right)\left( D_{1}+D_{2} \right)>0$，$\omega_{4}^{*}-\omega_{1}^{*}<0$，i.e. $\omega_{4}^{*}<\omega_{1}^{*}$; when $32\beta\eta\varphi_{m}\left( \beta+\varphi_{r} \right)C_{4}-\gamma^{2}\left( \beta+2\varphi_{r} \right)\left( D_{1}+D_{2} \right)<0$, $\omega_{4}^{*}>\omega_{1}^{*}$.

Where $D_{1}=2\beta^{2}\left( 13\beta^{2}\varphi_{m} + 3\varphi_{r}\beta^{2} + 24\beta{\varphi_{m}}^{2} + 55\varphi_{m}\varphi_{r}\beta+ 64\varphi_{r}{\varphi_{m}}^{2} \right)$；$D_{2}={\varphi_{r}}^{2}\left( 17\beta^{3} + 134\beta^{2}\varphi_{m} + 10\beta^{2}\varphi_{r} + 112\beta{\varphi_{m}}^{2}+ 50\varphi_{m}\varphi_{r}\beta+ 32\varphi_{r}{\varphi_{m}}^{2} \right)$

Appendix F. Compare with the manufacturer's optimal expected utility

${U\left( \Pi_{m} \right)}_{2}^{*}-{U\left( \Pi_{m} \right)}_{1}^{*}=\frac{2\eta\varphi_{r}\left( \alpha-\beta c \right)^{2}\left( 16\eta\beta\left( \beta+\varphi_{r} \right)- \gamma^{2}\left( \beta+2\varphi_{r} \right) \right)}{A_{1}\left( 16\beta\eta-7\gamma^{2} \right)}$，because $16\eta\beta>{30\gamma}^{2}$, so ${U\left( \Pi_{m} \right)}_{2}^{*}>{U\left( \Pi_{m} \right)}_{1}^{*}$;

Similarly,

${U\left( \Pi_{m} \right)}_{2}^{*}-{U\left( \Pi_{m} \right)}_{4}^{*}=\frac{4\eta\varphi_{m}\left( \alpha-\beta c \right)^{2}\left( \beta+\varphi_{r} \right)^{2}\left( \beta+2\varphi_{r} \right)\left( 16\beta\eta\left( \beta+\varphi_{r} \right)C_{4}-\gamma^{2}\left( \beta+{2\varphi}_{r} \right)\left( 13\beta^{3} + 36\varphi_{r}\beta^{2} + 24\beta^{2}\varphi_{m} + 20\beta{\varphi_{r}}^{2} + 40\varphi_{r}\beta\varphi_{m} + 16\varphi_{m}{\varphi_{r}}^{2} \right) \right)}{A_{1}\left( 16\beta\eta{C_{4}}^{2}-\gamma^{2}C_{5}C_{6}\left( \beta+\varphi_{r} \right) \right)}>0$

${U\left( \Pi_{m} \right)}_{4}^{*}-{U\left( \Pi_{m} \right)}_{3}^{*}=\frac{2\beta\eta\varphi_{r}\left( \alpha-\beta c \right)^{2}\left( 16\beta\eta\left( \beta+{2\varphi}_{m} \right)C_{4}\left( 4\beta\varphi_{m} + 2\beta\varphi_{r}+ 6\varphi_{r}\varphi_{m} + \beta^{2} \right)-\gamma^{2}\left( \beta+{2\varphi}_{r} \right)\left( 128{\varphi_{m}}^{3}{\varphi_{r}}^{2} + 256\beta{\varphi_{m}}^{3}\varphi_{r}+ 128\beta^{2}{\varphi_{m}}^{3} + 192\beta{\varphi_{m}}^{2}{\varphi_{r}}^{2} + 320\beta^{2}{\varphi_{m}}^{2}\varphi_{r} + 128\beta^{3}{\varphi_{m}}^{2} + 76\beta^{2}\varphi_{m}{\varphi_{r}}^{2} + 110\beta^{3}\varphi_{m}\varphi_{r} + 36\beta^{4}\varphi_{m} + 4\beta^{3}{\varphi_{r}}^{2}+ 4\beta^{4}\varphi_{r}+ \beta^{5} \right) \right)}{B_{1}\left( 16\beta\eta{C_{4}}^{2}-\gamma^{2}C_{5}C_{6}\left( \beta+\varphi_{r} \right) \right)}>0$

${U\left( \Pi_{m} \right)}_{1}^{*}-{U\left( \Pi_{m} \right)}_{3}^{*}=\frac{4\eta\varphi_{m}\left( \alpha-\beta c \right)^{2}\left( 16\eta\beta\left( \beta+2\varphi_{m} \right) -\gamma^{2} \left( 13\beta- 24\varphi_{m} \right) \right)}{B_{1}\left( 16\beta\eta-7\gamma^{2} \right)}>0$;

${U\left( \Pi_{m} \right)}_{4}^{*}-{U\left( \Pi_{m} \right)}_{1}^{*}=\frac{-2\eta\left( \alpha-\beta c \right)^{2}\left( 16\beta\eta C_{4}\left( 2\beta^{2}\varphi_{m} - \varphi_{r}\beta^{2} - 2\beta{\varphi_{r}}^{2} + 4\varphi_{r}\beta\varphi_{m} + 2\varphi_{m}{\varphi_{r}}^{2} \right)-\gamma^{2}\left( \beta+{2\varphi}_{r} \right)D_{3} \right)}{\left( 16\beta\eta-7\gamma^{2} \right)\left( 16\beta\eta{C_{4}}^{2}-\gamma^{2}C_{5}C_{6}\left( \beta+\varphi_{r} \right) \right)}$; when$16\beta\eta C_{4}\left( 2\beta^{2}\varphi_{m} - \varphi_{r}\beta^{2} - 2\beta{\varphi_{r}}^{2} + 4\varphi_{r}\beta\varphi_{m} + 2\varphi_{m}{\varphi_{r}}^{2} \right)-\gamma^{2}\left( \beta+{2\varphi}_{r} \right)D_{3}>0, {U\left( \Pi_{m} \right)}_{4}^{*}-{U\left( \Pi_{m} \right)}_{1}^{*}<0$, i.e. ${U\left( \Pi_{m} \right)}_{4}^{*}<{U\left( \Pi_{m} \right)}_{1}^{*}$; when$16\beta\eta C_{4}\left( 2\beta^{2}\varphi_{m} - \varphi_{r}\beta^{2} - 2\beta{\varphi_{r}}^{2} + 4\varphi_{r}\beta\varphi_{m} + 2\varphi_{m}{\varphi_{r}}^{2} \right)-\gamma^{2}\left( \beta+{2\varphi}_{r} \right)D_{3}<0$, ${U\left( \Pi_{m} \right)}_{4}^{*}>{U\left( \Pi_{m} \right)}_{1}^{*}$.

Where $D_{3}=32{\varphi_{r}}^{3}{\varphi_{m}}^{2} + 112\beta{\varphi_{m}}^{2}{\varphi_{r}}^{2} + 128\beta^{2}{\varphi_{m}}^{2}\varphi_{r} + 48\beta^{3}{\varphi_{m}}^{2} + 36\beta\varphi_{m}{\varphi_{r}}^{3} + 106 \beta^{2}\varphi_{m}{\varphi_{r}}^{2} + 96\beta^{3}\varphi_{m}\varphi_{r} + 26\beta^{4}\varphi_{m} - 4 \beta^{2}{\varphi_{r}}^{3} - 4\beta^{3}{\varphi_{r}}^{2} - \beta^{4}\varphi_{r}$

Appendix G Compare with the retailer’s optimal expected utility

${U\left( \Pi_{r} \right)}_{3}^{*}-{U\left( \Pi_{r} \right)}_{1}^{*}=\frac{64\beta\eta^{2}\varphi_{m}\left( \alpha-\beta c \right)^{2}\left( 256\beta^{2}\eta^{2}\left( \beta+3\varphi_{m} \right)\left( \beta+2\varphi_{m} \right)^{2}-\gamma^{2}\left( \beta+4\varphi_{m} \right)\left( 128\eta\beta^{3} - 7\beta^{2}\gamma^{2}+ 512\eta\beta^{2}\varphi_{m} - 41\beta\gamma^{2}\varphi_{m} + 512\eta\beta{\varphi_{m}}^{2} - 52\gamma^{2}{\varphi_{m}}^{2} \right) \right)}{{B_{1}}^{2}\left( 16\beta\eta-7\gamma^{2} \right)^{2}}$, because $16\eta\beta>{30\gamma}^{2}$, and we know that by sorting it out $256\beta^{2}\eta^{2}\left( \beta+3\varphi_{m} \right)\left( \beta+2\varphi_{m} \right)^{2}-\gamma^{2}\left( \beta+4\varphi_{m} \right)\left( 128\eta\beta^{3} - 7\beta^{2}\gamma^{2}+ 512\eta\beta^{2}\varphi_{m} - 41\beta\gamma^{2}\varphi_{m} + 512\eta\beta{\varphi_{m}}^{2} - 52\gamma^{2}{\varphi_{m}}^{2} \right)>0$, so ${U\left( \Pi_{r} \right)}_{3}^{*}>{U\left( \Pi_{r} \right)}_{1}^{*}$.

Similarly,

${U\left( \Pi_{r} \right)}_{3}^{*}-{U\left( \Pi_{r} \right)}_{4}^{*}=\frac{16\beta\eta^{2}\varphi_{r}\left( \alpha-\beta c \right)^{2}\left( 256\beta^{2}\eta^{2}\left( \beta+{2\varphi}_{m} \right)^{2}\left( \beta+\varphi_{r} \right){C_{4}}^{2}\left( 64{\varphi_{m}}^{4}{\varphi_{r}}^{2} + 128\beta{\varphi_{m}}^{2}\varphi_{r} + 64\beta^{2}{\varphi_{m}}^{4} + 160\beta{\varphi_{m}}^{3}{\varphi_{r}}^{2} + 288\beta^{2}{\varphi_{m}}^{3}\varphi_{r} + 132\beta^{2}{\varphi_{m}}^{2}{\varphi_{r}}^{2} + 204\beta^{3}{\varphi_{m}}^{2}\varphi_{r} + 76\beta^{4}{\varphi_{m}}^{2} + 40\beta^{3}\varphi_{m}{\varphi_{r}}^{2} + 52\beta^{4}\varphi_{m}\varphi_{r} + 16\beta^{5}\varphi_{m} + 4\beta^{4}{\varphi_{r}}^{2} + 4\beta^{5}\varphi_{r} + \beta^{6} \right) \right)}{{B_{1}}^{2}\left( 16\beta\eta{C_{4}}^{2}-\gamma^{2}\left( \beta+\varphi_{r} \right) \right)^{2}{C_{5}}^{2}{C_{6}}^{2}}>0$;

${U\left( \Pi_{r} \right)}_{1}^{*}-{U\left( \Pi_{r} \right)}_{2}^{*}=\frac{16\beta\eta^{2}\varphi_{r}\left( \alpha-\beta c \right)^{2}\left( 256\beta^{2}\eta^{2}\left( \beta+\varphi_{r} \right)^{3}-\gamma^{2}\left( 416\eta\beta^{4} - 133\beta^{3}\gamma^{2}+ 1216\eta\beta^{3}\varphi_{r}- 421\beta^{2}\gamma^{2}\varphi_{r}+ 1184\eta\beta^{2}{\varphi_{r}}^{2} - 431\beta\gamma^{2}{\varphi_{r}}^{2} + 384\eta\beta{\varphi_{r}}^{3}- 144\gamma^{2}{\varphi_{r}}^{3} \right) \right)}{{{A_{1}}^{2}\left( 16\beta\eta-7\gamma^{2} \right)}^{2}}>0$;

${U\left( \Pi_{r} \right)}_{4}^{*}-{U\left( \Pi_{r} \right)}_{2}^{*}=\frac{64\beta^{2}\eta^{2}{\varphi_{m}\left( \alpha-\beta c \right)}^{2}\left( \beta+\varphi_{r} \right)^{4}\left( 256\beta^{2}\eta^{2}\left( \beta+\varphi_{r} \right)^{2}{C_{4}}^{2}\left( 3\beta\varphi_{m} + 2\beta\varphi_{r}+ 3\varphi_{m}\varphi_{r}+ \beta^{2} \right)-\gamma^{2}\left( \beta+2\varphi_{r} \right)\left( 4\beta\varphi_{m} + 2\beta\varphi_{r}+ 4\varphi_{m}\varphi_{r} +\beta^{2} \right) \right)}{{A_{1}}^{2}\left( 16\beta\eta{C_{4}}^{2}-\gamma^{2}\left( \beta+\varphi_{r} \right) \right)^{2}{C_{5}}^{2}{C_{6}}^{2}}>0$;

${U\left( \Pi_{r} \right)}_{4}^{*}-{U\left( \Pi_{r} \right)}_{1}^{*}=\frac{16\beta\eta^{2}\left( \alpha-\beta c \right)^{2}\left( 256\beta^{2}\eta^{2}{C_{4}}^{2}D_{4}-\gamma^{2}D_{5} \right)}{\left( 16\beta\eta-7\gamma^{2} \right)^{2}\left( 16\beta\eta{C_{4}}^{2}-\gamma^{2}\left( \beta+\varphi_{r} \right) \right)^{2}{C_{5}}^{2}{C_{6}}^{2}}$，when $256\beta^{2}\eta^{2}{C_{4}}^{2}D_{4}-\gamma^{2}D_{5}>0$, ${U\left( \Pi_{r} \right)}_{4}^{*}-{U\left( \Pi_{r} \right)}_{1}^{*}>0$, i.e., ${U\left( \Pi_{r} \right)}_{4}^{*}>{U\left( \Pi_{r} \right)}_{1}^{*}$; when $256\beta^{2}\eta^{2}{C_{4}}^{2}D_{4}-\gamma^{2}D_{5}<0$, ${U\left( \Pi_{r} \right)}_{4}^{*}<{U\left( \Pi_{r} \right)}_{1}^{*}$.

Where $D_{4}=\left( \beta+\varphi_{r} \right)\left( 4\beta{\varphi_{m}}^{2}{\varphi_{r}}^{2}- 4{\varphi_{m}}^{2}{\varphi_{r}}^{3} + 20\beta^{2}{\varphi_{m}}^{2}\varphi_{r} + 12\beta^{3}{\varphi_{m}}^{2} - 8\beta\varphi_{m}{\varphi_{r}}^{3} - 4\beta^{2}\varphi_{m}{\varphi_{r}}^{2}+ 8\beta^{3}\varphi_{m}\varphi_{r} + 4\beta^{4}\varphi_{m}- 4\beta^{2}{\varphi_{r}}^{3}- 4\beta^{3}{\varphi_{r}}^{2}- \beta^{4}\varphi_{r} \right)$

$D_{5}=\left( 4\beta\varphi_{m} + 2\beta\varphi_{r}+ 4\varphi_{m}\varphi_{r} + \beta^{2} \right)\left( 32\beta\eta{C_{4}}^{2}\left( 16\varphi_{m}\beta^{3} - 13\beta^{3}\varphi_{r} - 38\beta^{2}{\varphi_{r}}^{2} + 12\varphi_{m}\beta^{2}\varphi_{r} - 24\beta{\varphi_{r}}^{3} - 20\varphi_{m}\beta{\varphi_{r}}^{2} - 16\varphi_{m}{\varphi_{r}}^{3} \right)-\gamma^{2}\left( 4\beta\varphi_{m} + 2\beta\varphi_{r}+ 4\varphi_{m}\varphi_{r} + \beta^{2} \right)\left( - 256{\varphi_{m}}^{2}{\varphi_{r}}^{6} - 1340\beta{\varphi_{m}}^{2}{\varphi_{r}}^{5} - 2732\beta^{2}{\varphi_{m}}^{2}{\varphi_{r}}^{4} - 2648\beta^{3}{\varphi_{m}}^{2}{\varphi_{r}}^{3} - 1128\beta^{4}{\varphi_{m}}^{2}{\varphi_{r}}^{2} - 76\beta^{5}{\varphi_{m}}^{2}\varphi_{r}+ 52\beta^{6}{\varphi_{m}}^{2}- 768\beta\varphi_{m}{\varphi_{r}}^{6} - 3576\beta^{2}\varphi_{m}{\varphi_{r}}^{5} - 6428\beta^{3}\varphi_{m}{\varphi_{r}}^{4} - 5504\beta^{4}\varphi_{m}{\varphi_{r}}^{3} - 2168\beta^{5}\varphi_{m}{\varphi_{r}}^{2} - 256\beta^{6}\varphi_{m}\varphi_{r} + 28\beta^{7}\varphi_{m}- 576\beta^{2}{\varphi_{r}}^{6} - 2300\beta^{3}{\varphi_{r}}^{5} - 3552\beta^{4}{\varphi_{r}}^{4} - 2647\beta^{5}{\varphi_{r}}^{3} - 953\beta^{6}{\varphi_{r}}^{2}- 133\beta^{7}\varphi_{r} \right) \right)$
